# Integration of transcriptomics and machine learning to explore inflammatory characteristics and key oxidative stress–related molecules in nasal polyps

**DOI:** 10.3389/fimmu.2026.1879295

**Published:** 2026-08-26

**Authors:** Yuelong Gu, Ru Tang, Zhihan Liu, Jiayao Zhou, Ying Zhu, Song Mao, Zhipeng Li, Weitian Zhang, Hai Lin

**Affiliations:** 1 Department of Otolaryngology-Head and Neck Surgery & Allergy Center, Shanghai Sixth People’s Hospital Affiliated to Shanghai Jiao Tong University School of Medicine, Shanghai, China; 2 Otolaryngological Institute, Shanghai Jiao Tong University, Shanghai, China; 3 Shanghai Key Laboratory of Sleep Disordered Breathing, Shanghai, China

**Keywords:** eosinophils, inflammatory response, machine learning, nasal polyps, oxidative stress, weighted gene co-expression network analysis

## Abstract

**Background:**

Chronic rhinosinusitis with nasal polyps (CRSwNP) is a chronic inflammatory disease of the upper airway driven by diverse inflammatory cells and mediators. This study aimed to characterize the inflammatory landscape of CRSwNP and to identify and validate oxidative stress-related key genes in CRSwNP.

**Methods:**

RNA sequencing of nasal mucosal tissues was performed in 22 subjects, including 8 controls, 5 patients with non-eosinophilic CRSwNP (neCRSwNP), and 9 patients with eCRSwNP. Differential expression analysis was conducted using DESeq2 R package. Enrichment analysis, Ingenuity Pathway Analysis (IPA),weighted gene co-expression network analysis (WGCNA) and CIBERSORT was performed. Differentially expressed genes (DEGs), eosinophilic inflammation-associated module genes, and oxidative stress-related genes were intersected to generate candidate genes, followed by protein-protein interaction (PPI) network analysis. Six machine learning models were constructed using the candidate genes. SHapley Additive exPlanations (SHAP) were used to interpret feature contributions, and receiver operating characteristic (ROC) curves were used to assess discriminatory performance. External single-cell transcriptomic datasets and nasal tissue samples were used for validation.

**Results:**

Enrichment analyses showed that eCRSwNP was prominently associated with immune-inflammatory responses and oxidative stress-related pathways. WGCNA identified four modules closely related to eosinophilic inflammation, and five oxidative stress-related core signature genes were ultimately selected: HIF1A, RAC2, SELP, NCF2, and NCF4. ROC analysis demonstrated good discriminatory ability for all five genes. SHAP analyses indicated consistent directions of effects and feature-prioritization patterns across algorithms. External validation and nasal tissue sample experiments confirmed the overall upregulation of these genes in eCRSwNP. Cell-type localization analyses indicated that NCF2, NCF4, and RAC2 were mainly derived from myeloid cells, and SELP was predominantly localized to vascular endothelial cells, and HIF1A was broadly expressed within inflammatory cell infiltration area.

**Conclusion:**

By integrating transcriptomic analysis with multi-algorithm machine learning, we identified and validated HIF1A, RAC2, SELP, NCF2, and NCF4 as oxidative stress-related core signature genes in eCRSwNP and confirmed their elevated expression and cell-type-specific localization in eCRSwNP tissues. These genes may contribute to reactive oxygen species-associated inflammatory processes and represent candidate molecular signatures for eCRSwNP endotyping and further mechanistic investigation.

## 1 Introduction

Chronic rhinosinusitis with nasal polyps (CRSwNP) is a chronic inflammatory disease characterized by persistent inflammation of nasal and sinus mucosa, tissue edema and polypoid hyperplasia. Its main symptoms include persistent nasal congestion, rhinorrhea, olfactory dysfunction, headache and facial pain, which seriously affect the quality of life and social function of patients (1). The pathogenesis of CRSwNP is complex, involving numerous factors such as genetic susceptibility, dysfunction of the epithelial barrier, microbial imbalance, and abnormal immune-inflammatory responses (2). The pathological features of CRSwNP mainly involve local inflammation of the nasal mucosa and excessive activation of the immune response. These reactions are jointly participated in by numerous structural cells (including epithelial cells and fibroblasts), immune cells (including lymphocytes, neutrophils, and eosinophils, etc.) and biological mediators (including cytokines and chemokines, etc.) (3, 4).

CRSwNP can be classified into eosinophilic endotype and non-eosinophilic endotype based on the quantity of eosinophils in nasal mucosa tissues (1). Considerable data indicate that eosinophilic CRSwNP (eCRSwNP) is frequently associated with type 2 inflammatory responses including increased expression of IL-4, IL-5 and IL-13, a higher level of inflammatory burden with continuous release of pro-inflammatory mediators and chemokines, leading to a poorer clinical prognosis, while non-eosinophilic CRSwNP (neCRSwNP) is associated with non-type 2 inflammatory responses (5, 6). However, the prevalence and inflammatory patterns of Type 2 CRSwNP vary considerably across different geographic populations (7). In particular, several studies have demonstrated important differences between Caucasian and Asian CRSwNP cohorts, including a historically higher prevalence of non-Type 2 (Type 1/Type 3) inflammation in Asian patients and the more recently described shift toward Type 2 inflammatory patterns (8, 9). According to the EPOS 2020 consensus (1), intranasal and short-course oral corticosteroids form first-line treatment, but many patients fail maximal medical therapy and need second-line alternatives. Interleukin-5 (IL-5) is a master cytokine governing eosinophil survival and activation, making anti-IL-5 monoclonal antibodies core targeted biologics for eosinophilic CRSwNP. Four anti-IL-5 monoclonal antibodies yield inconsistent symptom benefits in subgroup meta-analyses of randomized controlled trials (10). Such efficacy gaps reveal marked pathological heterogeneity of CRSwNP, including variable type 2 inflammation and mixed inflammatory phenotypes across populations. These divergent therapeutic responses clarify CRSwNP pathogenesis and validate precision biotherapy tailored to individual inflammatory endotypes.

Oxidative stress is a pathophysiological state in which reactive oxygen species (ROS) production exceeds the capacity of cellular antioxidant defense systems, resulting in disrupted redox homeostasis. ROS comprise chemically distinct species, including superoxide anion (O_2_•^−^), hydrogen peroxide (H_2_O_2_), and hydroxyl radicals (•OH); although physiological levels of ROS participate in cellular signaling, excessive or sustained accumulation can directly cause lipid peroxidation, protein oxidation modification, and DNA damage, damaging cell structure and normal functions, and subsequently inducing tissue damage and cell apoptosis (11, 12). Oxidative stress and inflammatory response have a close bidirectional positive regulatory relationship (13). Excessive ROS can activate classic inflammatory signaling pathways, significantly upregulate the expression of pro-inflammatory factors such as TNF-α, IL-6, and IL-1β, directly initiating and amplifying the inflammatory cascade. In chronic inflammation, major intracellular ROS sources include the mitochondrial electron transport chain and nicotinamide adenine dinucleotide phosphate (NADPH) oxidases (14). In activated inflammatory cells, the NADPH oxidase 2 (NOX2) complex primarily generates superoxide, which can be converted into H_2_O_2_ and drive redox-sensitive inflammatory signaling, thereby amplifying oxidative stress and inflammation (14, 15). Oxidative stress governs diverse disease outcomes and falls into two distinct categories: physiological oxidative eustress, where hydrogen peroxide and superoxide anion act as vital redox signaling messengers, and pathological oxidative distress triggered by excess oxidants. Disrupted redox balance drives biomolecular damage and aberrant signaling, contributing to cardiovascular disorders, neurodegeneration, cancer, fibrosis and inflammatory conditions (16). Subcellular oxidant production, environmental lifelong exposures and inter-organelle redox crosstalk jointly shape pathological phenotypes. Distinguishing eustress from distress via specific biomarkers is critical for developing precise redox-targeted therapies.

Current studies have shown that oxidative stress is closely related to the chronic inflammatory process of CRSwNP, and it has a highly specific association with eCRSwNP (17, 18). In the inflammatory microenvironment dominated by eosinophils, activated inflammatory cells can release a large amount of ROS, disrupting the local oxidative-antioxidative system balance of the nasal mucosa and inducing significant oxidative stress damage (19); while the excessive accumulation of ROS can further activate inflammatory signaling pathways, promoting the expression and release of type 2 pro-inflammatory factors such as IL-4, IL-5, and IL-13, continuously recruiting and activating eosinophils, aggravating mucosal epithelial damage, tissue edema, and polyp remodeling, forming a pathological vicious cycle of eosinophilic inflammation-oxidative stress, and ultimately promoting the occurrence, development, and recurrence of nasal polyps (19, 20). In-depth elucidation of the key molecules of oxidative stress in eCRSwNP can provide new theoretical basis for the mechanism research and targeted intervention of this refractory endotype of nasal polyps.

In recent years, with the rapid development of high-throughput sequencing technology, disease-related differentially expressed genes (DEGs) can be efficiently screened at the transcriptome level, providing an important data foundation for analyzing the molecular mechanisms of diseases and exploring potential regulatory targets. However, traditional methods for DEGs screening often have shortcomings such as redundant results, high false positive rates, and difficulty in precisely identifying core functional genes and capturing coordinated biological pathways, which greatly limit the subsequent research on mechanisms (21, 22). Weighted gene co-expression network analysis (WGCNA) can partly address this limitation by identifying modules of co-regulated genes, yet CRSwNP studies using this approach remain limited and often lack integration with well-defined endotypes and clinically relevant inflammatory traits (23–25). In parallel, machine learning algorithms possess excellent capabilities in reducing high-dimensional data, feature selection, and pattern recognition, which can effectively overcome the deficiencies of traditional transcriptome analysis. Combining the initial screening of transcriptome DEGs with the selection of machine learning features can simplify the set of DEGs while precisely selecting candidate genes with high discriminative value and core regulatory functions, improving the accuracy and reliability of key target screening, and providing a new research idea and data support for in-depth exploration of the molecular mechanism of CRSwNP.

Based on these considerations, the present study was designed to systematically characterize inflammatory and oxidative stress–related molecular features of CRSwNP, with a particular focus on eCRSwNP. Using bulk RNA-sequencing data from control mucosa tissues, neCRSwNP tissues, and eCRSwNP tissues, we first defined transcriptomic differences through DEGs analysis and multiple pathway-enrichment approaches. We then applied WGCNA to identify co-expression modules associated with eosinophilic inflammatory traits and integrated these modules with oxidative stress–related gene sets to prioritize biologically relevant candidates. To improve the robustness of feature selection, we further used multiple machine-learning algorithms combined with SHapley Additive exPlanations (SHAP)-based interpretation to identify stable oxidative stress–related signature genes. Finally, we incorporated immune infiltration analysis, public single-cell RNA sequencing (scRNA-seq) data, and tissue-level validation by qRT-PCR, immunohistochemistry, and immunofluorescence to define the expression patterns, cellular origins, and pathological relevance of the identified genes. Through this multi-level framework, we aimed to establish an interpretable molecular framework for understanding eCRSwNP heterogeneity and to provide candidate biomarkers and mechanistic clues for future precision stratification and targeted intervention.

## 2 Methods

### 2.1 Study design and workflow

This study aimed to systematically integrate various screening strategies, including bulk transcriptome DEGs analysis, gene interaction regulatory network analysis, machine learning feature selection and screening, external validation using public single-cell sequencing data sets, and experimental verification using clinical nasal mucosa tissue samples. We conducted multi-level screening and systematic validation of key oxidative stress characteristic genes closely related to the inflammatory process of eCRSwNP. First, we collected nasal mucosa tissue samples from healthy control groups, neCRSwNP and eCRSwNP for RNA sequencing. Second, various bioinformatics analysis methods including DEGs screening, functional enrichment analysis, ingenuity pathway analysis (IPA), WGCNA, immune infiltration analysis, and protein-protein interaction (PPI) network analysis were performed in sequence. Then, through the analysis of Venn intersection, the DEGs, genes involved in inflammatory regulation modules, and genes with characteristics of oxidative stress were cross-screened to initially obtain the key oxidative stress genes highly related with eCRSwNP. To further eliminate redundant genes, prioritize candidate genes with higher discriminatory potential and improve feature selection efficiency, six different algorithm principles-based machine learning models were constructed to objectively score the importance and prioritize the selected characteristic genes. This further compressed and screened out high-identification core genes. Finally, based on the publicly available scRNA-seq data sets, the cell distribution and expression characteristics of the key genes were analyzed at the single-cell level, and tissue experiments were conducted using clinical nasal mucosa tissue samples to verify the expression patterns and distribution perspectives of the candidate genes.

### 2.2 Patients and sample collection

Patients who underwent functional endoscopic sinus surgery (FESS) in the Department of Otolaryngology-Head and Neck Surgery between August 2023 and July 2024 were prospectively enrolled. The cohort included 22 subjects: 8 controls, 9 patients with eCRSwNP, and 5 patients with neCRSwNP. CRSwNP, eCRSwNP and neCRSwNP were diagnosed according to the European Position Paper on Rhinosinusitis and Nasal Polyps 2020 (EPOS 2020) criteria based on clinical presentation, nasal endoscopy, sinus computed tomography and eosinophils per high power field (1). The inclusion criteria are listed below (1): Participants were divided into two groups: adult patients with CRSwNP diagnosed per the EPOS 2020 diagnostic criteria, and control individuals free of chronic inflammatory sinonasal disorders (2); no oral/nasal glucocorticoids, antibiotics, or biologics had been used by any participant within four weeks before surgery. Control nasal mucosa tissue samples were obtained from inferior turbinate mucosa of patients undergoing septoplasty for deviated nasal septum, whereas CRSwNP samples were collected from nasal polyp tissues during FESS. Patients with established antrochoanal polyps, immunodeficiency, coagulation disorder, cystic fibrosis, fungal sinusitis or a history of aspirin sensitivity were excluded. In addition, previous sinonasal surgery history has been recorded, and 4 patients with eCRSwNP and 1 patient with neCRSwNP underwent revision surgery, while the remaining patients had no history of previous nasal surgery. Detailed characteristics of enrolled subjects are listed in **Supplementary Table 1**. Fresh tissue samples were collected for RNA sequencing. This study was approved by the Ethical Committee of Shanghai Sixth People’s Hospital Affiliated to Shanghai Jiao Tong University School of Medicine, and all subjects provided informed consent. More information of all the subjects was included in the **Supplementary Material**.

### 2.3 Bulk RNA sequencing and preprocessing

Total RNA extracted from frozen tissues were used for bulk RNA sequencing. After RNA quality assessment, poly(A)-enriched libraries were constructed and sequenced on an Illumina NovaSeq 6000 platform using paired-end 150-bp reads. Raw FASTQ files were subjected to sequencing-quality assessment, adapter removal, and low-quality read filtering using FastQC and Trimmomatic. The resulting clean reads were aligned to the human reference genome GRCh38 using HISAT2, and gene-level raw read counts were quantified using featureCounts with the corresponding human gene annotation file. Raw integer read counts were used as input for differential expression analysis with DESeq2. Library-size and composition differences among samples were normalized internally using the standard DESeq2 median-of-ratios size-factor method, followed by gene-wise dispersion estimation and negative binomial generalized linear modeling. FPKM values were used for expression visualization, WGCNA, and immune-cell infiltration analysis. Principal component analysis (PCA) was performed after variance-stabilizing transformation to assess global transcriptomic differences among groups. Detailed library-construction procedures, software versions, parameter settings, and quality-control criteria are provided in the **Supplementary Material**.

### 2.4 DEGs and functional enrichment analyses

DEGs were identified using the DESeq2 R package (v1.38.3). Three pairwise comparisons were performed: control versus eCRSwNP, control versus neCRSwNP, and neCRSwNP versus eCRSwNP. In each comparison, the first group served as the reference group. Genes with adjusted *P* value < 0.05 and |log2 fold change| >= 1 were considered differentially expressed. “Clusterprofiler” R package (v4.18.4) was used to perform functional interpretation of DEGs including Gene Ontology (GO), Kyoto Encyclopedia of Genes and Genomes (KEGG), and Gene Set Enrichment Analysis (GSEA) (26). IPA was further used to predict canonical pathways and disease and function activation states. Enrichment pathways of DEGs were generated based on the Ingenuity Pathway Knowledge Data Base. Additional details are provided in the **Supplementary Material**.

### 2.5 Co-expression network analysis and immune infiltration analysis

To identify co-expression modules associated with eosinophilic inflammation, WGCNA was performed using the WGCNA package (v1.73) (27). Low-expression and low-variation genes were removed before network construction, and sample hierarchical clustering and connectivity analysis were used to identify potential outliers. The soft-thresholding power was selected using the pickSoftThreshold function as the lowest value achieving an approximately scale-free topology fit of R² = 0.8–0.9 while retaining reasonable mean connectivity; β = 11 was ultimately selected. A weighted adjacency matrix and topological overlap matrix were then constructed, and genes were hierarchically clustered using topological overlap dissimilarity, followed by dynamic tree cutting to identify co-expression modules. Module eigengenes were correlated with blood eosinophil percentage, blood eosinophil count, tissue eosinophil percentage, and tissue eosinophil count. Modules showing significant and directionally consistent positive associations across multiple eosinophilic traits were retained for downstream analysis. Given the limited cohort size, these module–trait associations were interpreted as exploratory. Immune cell infiltration was estimated using CIBERSORT (v1.04) with the LM22 reference matrix (28). Differences in immune-cell fractions among groups were assessed, and correlations between module eigengenes and immune-cell proportions were further examined. Detailed procedures and parameter settings are described in the **Supplementary Material**.

### 2.6 Candidate oxidative stress-related gene identification and PPI analysis

Oxidative stress-related genes were retrieved from the GeneCards database using the keyword “oxidative stress,” and genes with a Relevance Score >= 7 were retained. Candidate genes were defined as the intersection of three gene sets: DEGs from the control versus eCRSwNP comparison, genes from eosinophilic inflammation-associated WGCNA modules, and oxidative stress-related genes from GeneCards. PPI analysis was then performed using STRING, followed by Cytoscape visualization. Genes within the top 50% ranked by degree were retained for subsequent machine-learning analysis. Detailed procedures are provided in the **Supplementary Material**.

### 2.7 Machine-learning analysis and feature selection

Machine-learning analysis was performed for exploratory feature prioritization between eCRSwNP and control samples because of the limited sample size of the neCRSwNP group. Eighteen candidate genes derived from integrative transcriptomic and PPI screening were used as input features. Six machine-learning algorithms with different feature-selection or ranking properties were applied in parallel: least absolute shrinkage and selection operator (LASSO), ridge regression, elastic net, random forest, k-nearest neighbors (KNN), and support vector machine (SVM). These algorithms were used as exploratory tools to rank and prioritize candidate genes rather than to develop or validate a clinically applicable predictive classifier. Leave-one-out cross-validation (LOOCV) was used during algorithm-specific hyperparameter tuning because of the limited sample size; however, upstream transcriptomic and network-based candidate-gene screening had been performed using the full discovery cohort.

SHAP analysis was conducted to assess feature contribution and model interpretability. Single-gene receiver operating characteristic (ROC) analysis was then used to assess the discriminatory ability of the final signature genes. Detailed model specifications, tuning procedures, SHAP implementation, and visualization strategies are described in the **Supplementary Material**.

### 2.8 External scRNA-seq validation

An external public scRNA-seq dataset of nasal polyp tissues and control nasal mucosa (Genome Sequence Archive, GSA, HRA000772) was used for cellular validation. The dataset contained 22 nasal mucosal tissue samples, including 5 control nasal mucosal tissues and 11 nasal polyps (6 eCRSwNP and 5 neCRSwNP). Expression patterns of the identified genes were examined using uniform manifold approximation and projection (UMAP) feature plots, violin plots, dot plots, and heatmaps.

To assess coordinated activity, a five-gene module score was calculated using AddModuleScore. Pseudotime analysis was performed with Monocle3 (v1.3.4) on a stratified immune-cell subset to explore trajectory structure. In addition, correlations between the five-gene score and oxidative stress-related module scores were evaluated. Additional information can be found in the **Supplementary Material**.

### 2.9 Nasal tissue sample experiments

Clinical specimens utilized in the experiments comprised 53 nasal mucosal tissue samples: 16 healthy control nasal mucosal tissues and 37 nasal polyp tissues (20 eCRSwNP and 17 neCRSwNP). qRT-PCR was performed to validate the mRNA expression of the selected genes in nasal tissue samples. The primer sequences used in qRT-PCR was listed in **Supplementary Table 2**. Immunohistochemistry (IHC) was used to evaluate histological expression and distribution of targeted molecules, and double immunofluorescence (IF) was used to determine cellular localization by co-staining target proteins with lineage-specific markers. Experimental antibody information was listed in **Supplementary Table 3**. Additional information can be found in the **Supplementary Material**.

### 2.10 Heatmap visualization and clustering

Heatmaps were generated using the pheatmap package (v1.0.13). Where clustering was applied, hierarchical agglomerative clustering was used. The global DEG expression heatmap was clustered using Euclidean distance and complete linkage. Feature-importance and cell-type expression heatmaps were clustered using Pearson correlation-based distance (1 − r) and complete linkage. For WGCNA, genes were hierarchically clustered using topological overlap dissimilarity and average linkage, followed by module identification using the dynamic tree-cutting algorithm. Module-trait and module-immune-cell association heatmaps were displayed without additional clustering. Detailed clustering parameters for individual heatmaps are provided in the **Supplementary Methods**.

### 2.11 Statistical analysis

For transcriptomic analyses, all statistical analyses were performed in R v4.5.1. For experimental validation, statistical analyses and data visualization were performed using GraphPad Prism. Group comparisons were performed using the Mann-Whitney U test, or the Kruskal-Wallis test with appropriate *post hoc* correction. Categorical variables were compared using the chi-square test or Fisher’s exact test. Distributional assumptions using Shapiro-Wilk test were assessed prior to applying correlation analysis. If the data followed a normal distribution, Pearson correlation analysis was used for testing. If the data were not normally distributed, Spearman rank correlation analysis was applied for correlation analysis. We have added these in the Methods section. A two-sided *P* value < 0.05 was considered statistically significant.

## 3 Results

### 3.1 Transcriptomic profiling reveals distinct inflammatory and oxidative stress signatures in CRSwNP

**Figure 1:**
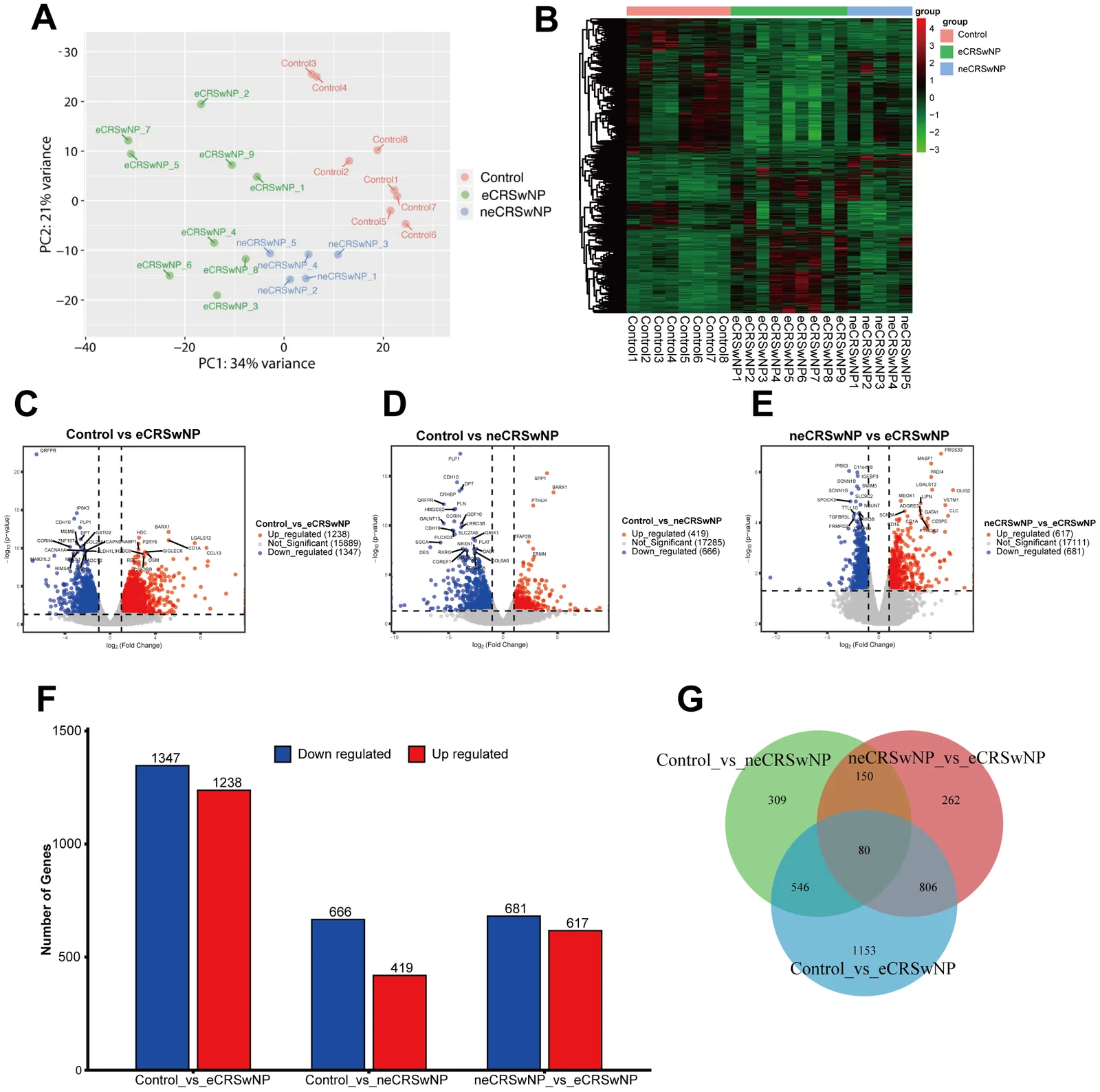
Global transcriptomic profiling reveals distinct expression patterns among Control, neCRSwNP, and eCRSwNP samples. **(A)** Principal component analysis of RNA-seq data. PCA scatter plot of transcriptomic data showing PC1 (x-axis, 34% variance explained) versus PC2 (y-axis, 21% variance explained). Dots denote individual samples. **(B)** Hierarchical clustering heatmap of DEGs across all samples. The color scale gradient from green through black to red corresponds to relative gene expression values ranging from −3 to 4 **(C–E)** Volcano plots of DEGs identified in pairwise comparisons: Control vs. eCRSwNP **(C)**, Control vs. neCRSwNP **(D)**, and neCRSwNP vs. eCRSwNP **(E)**. **(F)** Numbers of up- and downregulated DEGs in each comparison. **(G)** Venn diagram detailing the overlap of DEGs among comparisons. neCRSwNP, non-eosinophilic chronic rhinosinusitis with nasal polyps; eCRSwNP, eosinophilic chronic rhinosinusitis with nasal polyps; PC, principal component; DEGs, differentially expressed genes.

**Figure 2:**
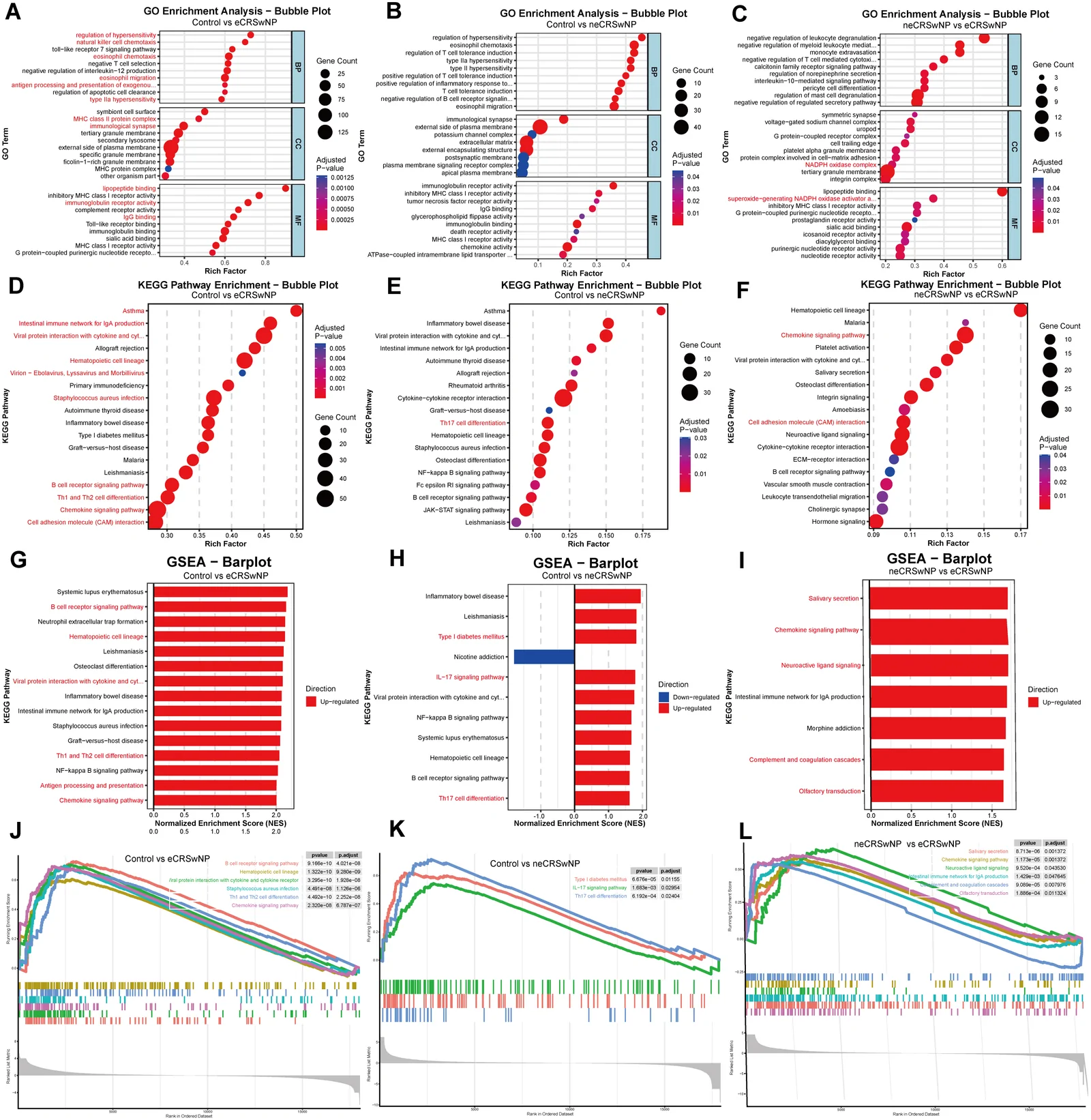
Functional enrichment analyses reveal specific inflammatory and oxidative stress-related pathways among CRSwNP endotypes. **(A–C)** GO enrichment bubble plots of DEGs in Control vs. eCRSwNP **(A)**, Control vs. neCRSwNP **(B)**, and neCRSwNP vs. eCRSwNP **(C)**. Bubble size corresponds to gene count, and color indicates adjusted *P*-value. **(D–F)** KEGG enrichment bubble plots for the three pairwise comparisons. **(G–I)** GSEA bar plots highlighting the top enriched KEGG pathways. **(J–L)** Representative GSEA enrichment plots for selected pathways across comparisons.

Transcriptomic profiling of 22 tissue samples (8 Control, 5 neCRSwNP, and 9 eCRSwNP specimens) revealed distinct gene expression patterns across the cohorts. PCA demonstrated clear group separation, with eCRSwNP exhibiting the most divergent expression profile (**Figure 1A**). Hierarchical clustering revealed distinct expression patterns among Control, eCRSwNP, and neCRSwNP samples (**Figure 1B**). In the comparison of control versus eCRSwNP, 2,585 DEGs were identified, including 1238 genes upregulated and 1347 genes downregulated in eCRSwNP relative to control. In the comparison of control versus neCRSwNP, 1,085 DEGs were identified, including 419 genes upregulated and 666 genes downregulated in neCRSwNP relative to control. In the comparison of neCRSwNP versus eCRSwNP, 1,298 DEGs were identified, including 617 genes upregulated and 681 genes downregulated in eCRSwNP relative to neCRSwNP (**Figures 1C–F**). The Venn diagram can visually display the distribution characteristics of the common and unique differentially expressed genes among the groups (**Figure 1G**), further confirming that the nasal mucosa tissues of eCRSwNP patients have a more pronounced transcriptomic dysregulation. To deeply explore the biological functions mediated by the DEGs, we conducted GO, KEGG, GSEA, and IPA enrichment analyses on the DEGs obtained from the three comparisons. The comparison between the control group and eCRSwNP showed that the GO functional categories were significantly enriched in the processes including hypersensitivity reaction regulation, leukocyte and eosinophil chemotaxis, antigen processing and presentation (**Figure 2A**); the KEGG pathway analysis revealed asthma, chemokine signaling pathways, cell adhesion molecule interactions, and immune defense pathways related to pathogen infection (**Figure 2D**), suggesting that eCRSwNP exhibited a predominantly type 2 inflammatory profile, accompanied by a large number of immune cell infiltration and recruitment in our cohort. In contrast, the comparison between the control group and neCRSwNP also indicated that the inflammatory response was activated, but its inflammatory characteristics had obvious heterogeneity, mainly enriched in cytokine-mediated immune responses, JAK-STAT signaling pathways, and IL-17-related inflammatory pathways (**Figures 2B, E**), suggesting a divergent inflammatory milieu. Notably, in the neCRSwNP vs. eCRSwNP comparison, enriched GO and KEGG terms predominantly associated with the regulation of myeloid cell activation, superoxide-generating NADPH oxidase activity, chemokine signaling, and cell adhesion molecule interactions (**Figures 2C, F**).

GSEA corroborated these observations at the transcriptome level. In the Control vs. eCRSwNP comparison, pathways related to B-cell receptor signaling, hematopoietic cell lineage, antigen processing and presentation, and chemokine signaling were prominently enriched (**Figures 2G, J**). In the Control vs. neCRSwNP comparison, IL-17 signaling emerged as the representative enriched pathway (**Figures 2H, K**). When comparing neCRSwNP to eCRSwNP, chemokine signaling and complement/coagulation-related pathways remained significantly enriched (**Figures 2I, L**). Furthermore, IPA demonstrated that eCRSwNP associated with a more robust activation of phagosome formation, pathogen-induced cytokine storm signaling, neutrophil degranulation, S100 family signaling, focal adhesion kinase signaling, and Th2 pathways; similar, albeit less intense, activation was observed in neCRSwNP. Disease and functional annotation indicated that eCRSwNP displayed broader activation of immune responses, cellular stress and injury, tissue remodeling, and neuroimmune-related signaling (**Supplementary Figure 1**; **Supplementary Tables 4**–**6**). Collectively, these multi-layered functional analyses indicate that eCRSwNP exhibits stronger inflammatory activation and more prominent oxidative stress-related dysregulation than neCRSwNP.

### 3.2 WGCNA identifies eosinophilic inflammation–associated gene modules in CRSwNP

**Figure 3:**
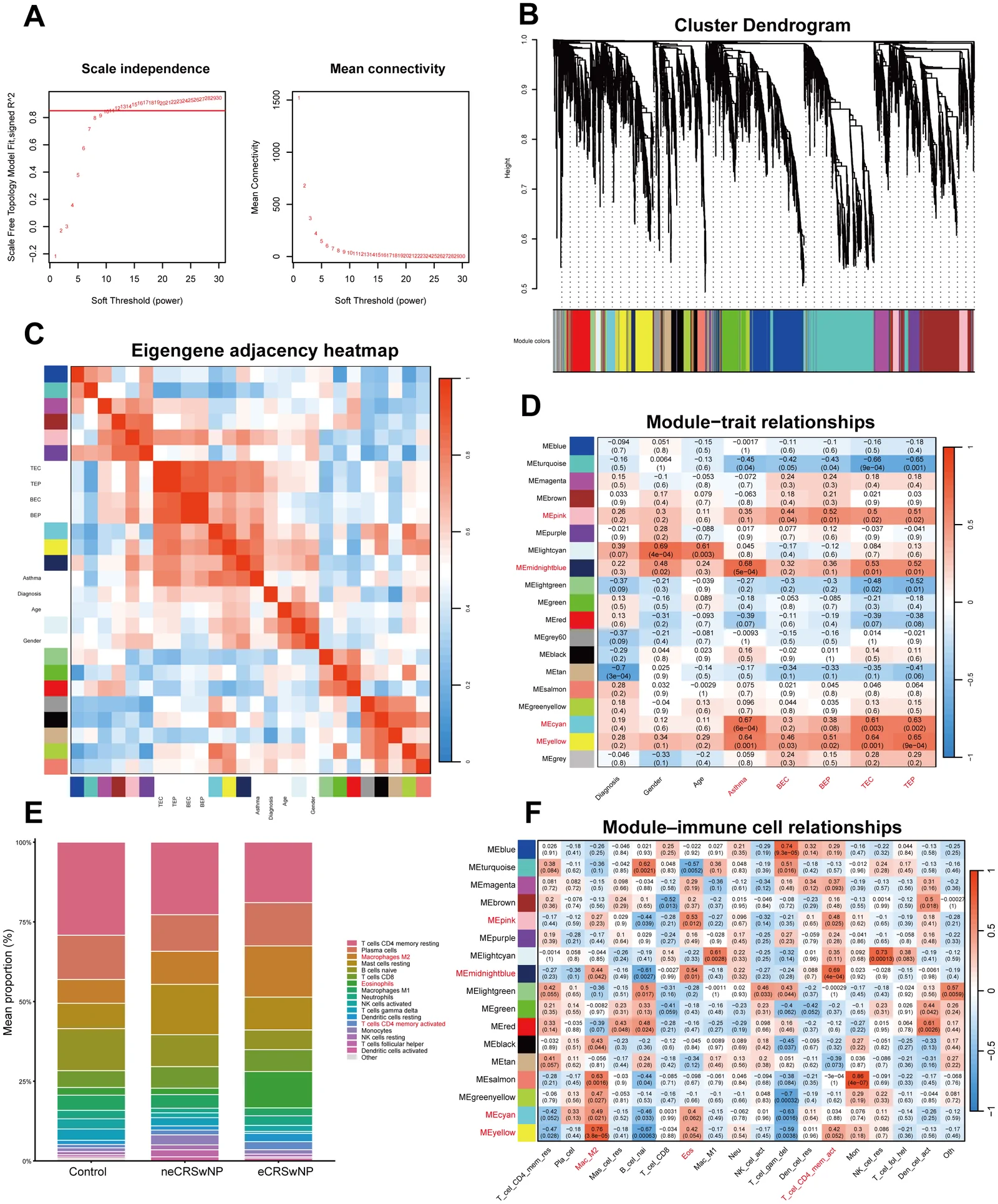
WGCNA and immune infiltration analysis identify eosinophilic inflammation-associated co-expression modules in CRSwNP. **(A)** Network topology analysis for soft-threshold selection, demonstrating scale-free topology fit (signed R²) and mean connectivity. **(B)** Hierarchical clustering dendrogram based on topological overlap. **(C)** Eigengene adjacency heatmap mapping relationships among module eigengenes and clinical traits. **(D)** Heatmap of module-trait correlations (Pearson coefficients and *P*-values shown in cells). **(E)** Stacked bar plot of mean immune cell subset proportions inferred by CIBERSORT. **(F)** Heatmap of correlations between module eigengenes and immune cell fractions. The color scale gradient from blue through white to red indicates that correlation coefficients covering inter-module associations, module–trait correlations, and module–immune cell correlations increase from low to high values. BEP, blood eosinophil percentage; BEC, blood eosinophil count; TEP, tissue eosinophil percentage; TEC, tissue eosinophil count.

To identify co-expression modules associated with eosinophilic inflammation, we constructed a weighted gene co-expression network using the DEGs and integrated it with clinical eosinophilic indices and immune infiltration profiles. A soft-thresholding power of β = 11 was selected to achieve an approximately scale-free topology (**Figure 3A**), and multiple co-expression modules were subsequently identified via hierarchical clustering based on topological overlap (**Figure 3B**). The analysis of module eigengene analysis and module–trait correlation mapping resulted in the identification of four key gene modules, namely ME yellow, ME cyan, ME pink, and ME midnight blue modules (**Figures 3C, D**). These modules all showed significant positive correlations with clinical indicators of eosinophilic inflammation, including asthma phenotype, tissue eosinophil percentage (TEP), tissue eosinophil count (TEC), peripheral blood eosinophil percentage (BEP), and peripheral blood eosinophil count (BEC).

The CIBERSORT immune infiltration analysis results indicated that there were significant differences in the immune microenvironment infiltration patterns among the three groups of samples; the eCRSwNP group was characterized mainly by increased infiltration of eosinophils and myeloid immune cells, while the control group and the neCRSwNP group had relatively higher proportions of various lymphocyte subpopulations (**Figure 3E**). The box plot analysis results were consistent with the immune infiltration trend: compared to the control group and the neCRSwNP group, the infiltration level of eosinophils in nasal mucosal tissue of the eCRSwNP group was significantly upregulated, while the infiltration proportions of naive B cells and resting memory CD4^+^T cells were significantly reduced (**Supplementary Figure 2**). Correlation analysis between module eigengenes and immune cell fractions further established that the four eosinophilic inflammation-associated modules positively correlated with characteristic immune cell populations enriched in eCRSwNP, particularly eosinophils, activated memory CD4^+^ T cells, and M2 macrophages (**Figure 3F**). Among these, MEyellow showed the strongest positive correlation with M2 macrophages, whereas MEmidnightblue displayed prominent positive correlations with eosinophils and activated memory CD4^+^ T cells. Together, these findings support MEyellow, MEcyan, MEpink, and MEmidnightblue as representative co-expression modules intimately linked to the eosinophilic inflammatory endotype of CRSwNP.

### 3.3 Integrative screening prioritizes oxidative stress–related candidate genes in eCRSwNP

**Figure 4:**
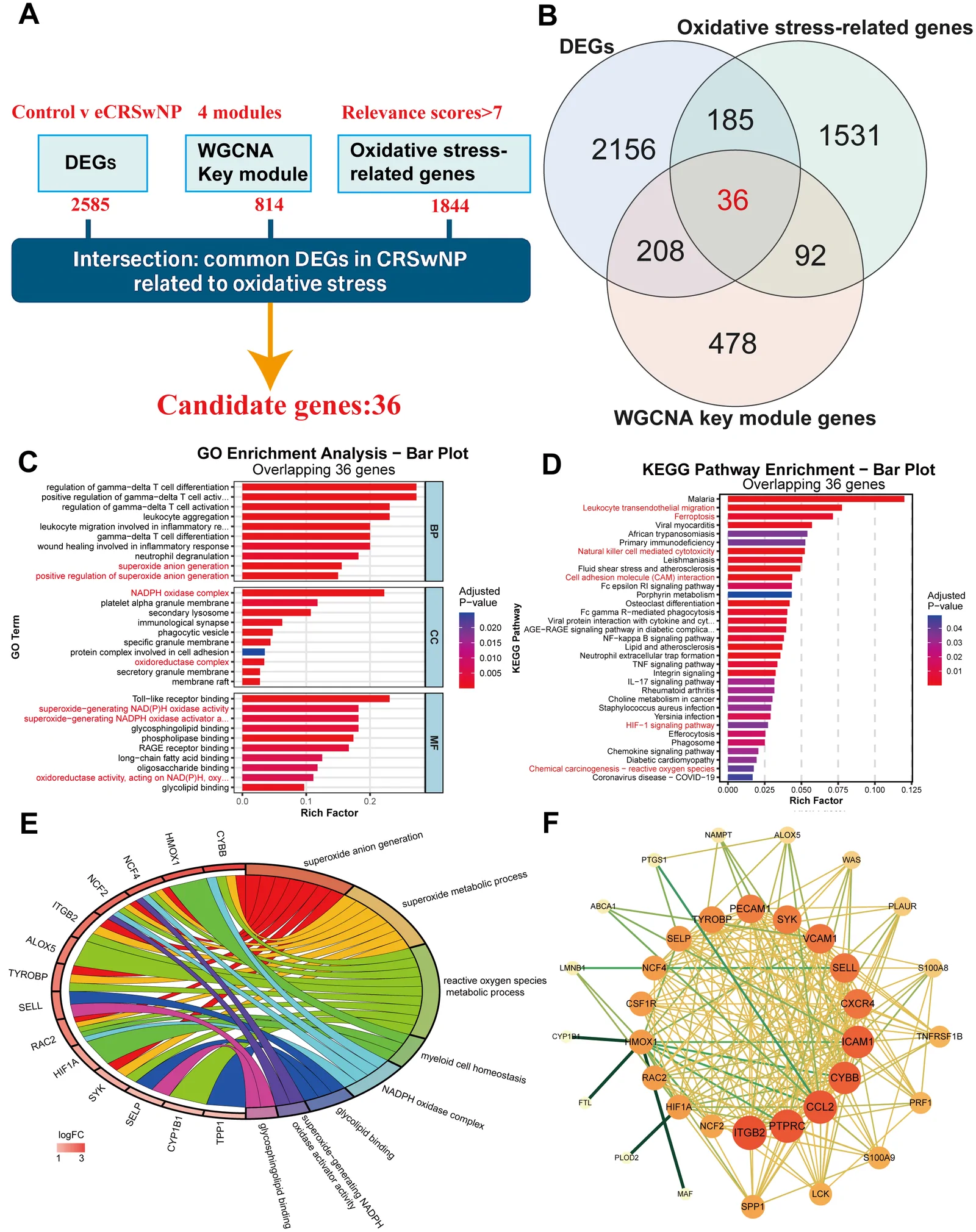
Integrative analysis identifies oxidative stress-related candidate genes in eCRSwNP. **(A)** Schematic workflow for candidate-gene identification. **(B)** Venn diagram showing the intersection of three gene sets, yielding 36 candidate genes. **(C)** GO enrichment bar plot of candidate genes (bar length indicates rich factor, color indicates adjusted *P*-value). **(D)** KEGG enrichment bar plot. **(E)** Chord plot mapping associations between candidate genes and enriched pathways. The left gene semicircle uses red gradient to denote logFC (darker red = higher upregulation). Each ribbon represents one gene-GO term pair. Ribbon bundle width for each GO term is inversely related to its enriched gene count: fewer genes correspond to wider ribbons. **(F)** PPI network of candidate genes. Node fill colour transitions from pale yellow to deep red to reflect node degree; deeper red nodes represent hub genes with more interaction partners. Two edge colours distinguish interaction evidence grades: green solid lines indicate experimentally validated high-confidence protein interactions, and golden-yellow solid lines represent moderate-confidence predicted interactions from the STRING database. Edge thickness is proportional to the combined confidence score of each pairwise protein interaction (thicker lines = stronger binding evidence).

Given the more pronounced oxidative stress-related pathway activation observed in eCRSwNP, we performed an integrative intersection analysis combining the DEGs from the control versus eCRSwNP comparison, the eosinophilic inflammation-associated WGCNA modules, and oxidative stress-related genes retrieved from GeneCards. This analysis identified 36 genes that were consistently upregulated in eCRSwNP: ABCA1, ALOX5, CCL2, CSF1R, CXCR4, CYBB, CYP1B1, FTL, HIF1A, HMOX1, ICAM1, ITGB2, LCK, LMNB1, MAF, NAMPT, NCF2, NCF4, PECAM1, PLAUR, PLOD2, PRF1, PTGS1, PTPRC, RAC2, S100A8, S100A9, SELL, SELP, SPP1, SYK, TNFRSF1B, TPP1, TYROBP, VCAM1, and WAS (**Figures 4A, B**). Among these genes, CYBB, NCF2, NCF4, and RAC2 are closely related to NADPH oxidase-mediated ROS generation, whereas CCL2, CXCR4, ICAM1, ITGB2, PECAM1, SELL, SELP, and VCAM1 are mainly involved in leukocyte recruitment, adhesion, and transendothelial migration.

Functional enrichment analysis of these 36 candidate genes reinforced their close association with oxidative stress and inflammatory responses. GO analysis revealed significant enrichment in superoxide anion generation, positive regulation of superoxide anion generation, and localization to the NADPH oxidase and oxidoreductase complexes. The enriched molecular functions primarily related to NADPH oxidase activity, superoxide-generating NADPH oxidase activity, NADPH oxidase activator activity, and oxidoreductase activity acting on NAD(P)H (**Figure 4C**). KEGG analysis further revealed enrichment in leukocyte transendothelial migration, cell adhesion molecules, ferroptosis, HIF-1 signaling, and chemical carcinogenesis–reactive oxygen species (**Figure 4D**); this suggests these genes participate not only in ROS generation and redox regulation, but also in inflammatory cell recruitment, adhesion, tissue infiltration, and stress-related tissue remodeling. The chord diagram analysis demonstrates that the majority of candidate genes are simultaneously enriched in functional entries associated with oxidative stress and immune inflammation, forming a highly coupled gene functional regulatory network (**Figure 4E**). Consistent with this finding, PPI analysis confirms the existence of close interactions among the 36 candidate genes, and identifies multiple core nodes with high connectivity within the network (**Figure 4F**). In this study, we ranked all nodes by connectivity and selected the top 50% of genes (18 in total) as core interaction genes for subsequent analysis. This screening strategy retained the completeness of the core biological regulatory network while further simplifying the candidate gene set.

### 3.4 Machine learning identifies five oxidative stress–related core genes in eCRSwNP

**Figure 5:**
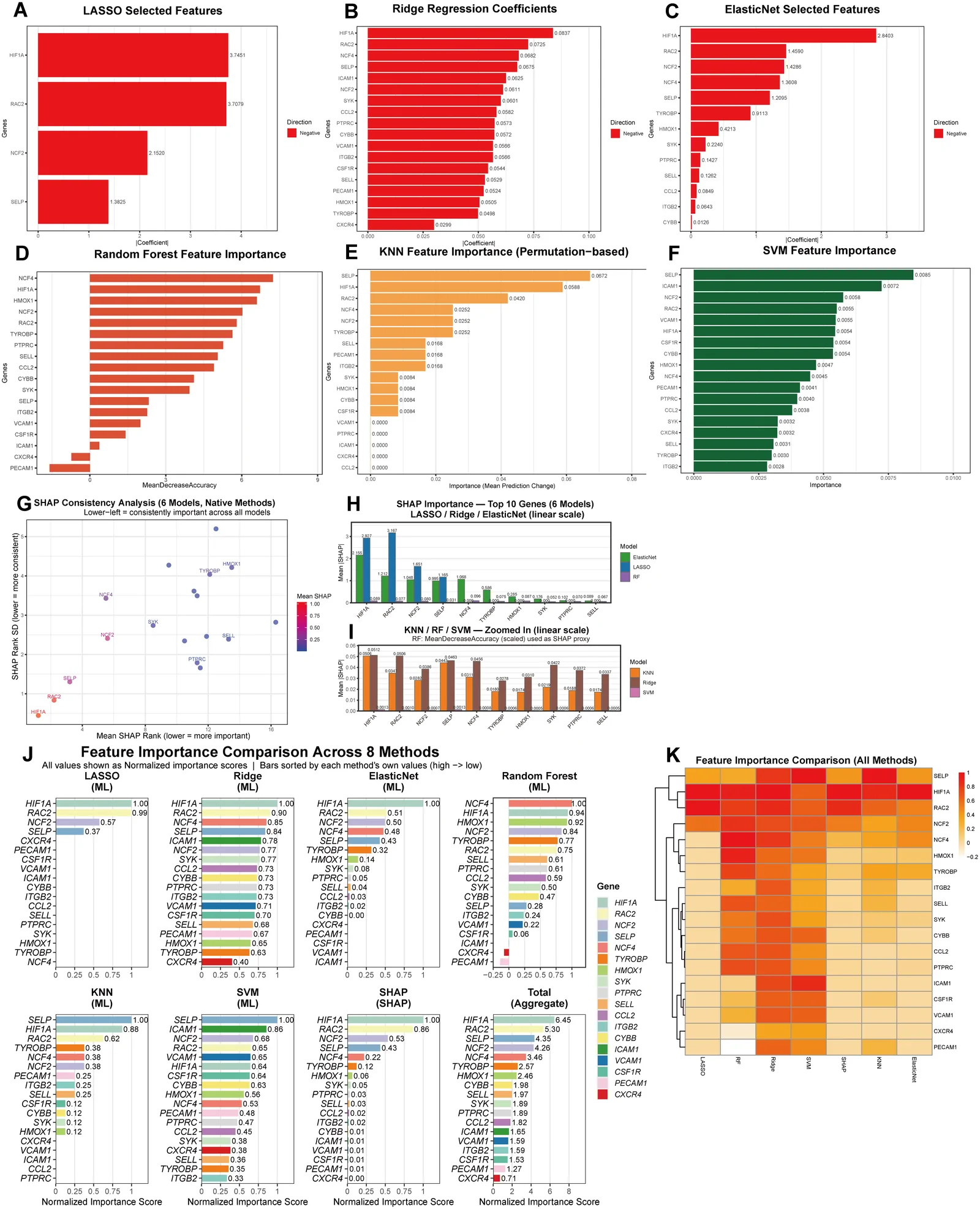
Feature-importance and SHAP-based analyses across six machine-learning models. **(A–F)** Feature importance in LASSO, Ridge, Elastic Net, random forest, KNN, and SVM models. **(G)** Scatter plot of SHAP consistency across models. **(H–I)** Comparison of mean SHAP values across models. **(J)** Integrated comparison of normalized feature importance across all methods, including the aggregated total score. **(K)** Heatmap mapping the relative importance of candidate genes across methods.

Six machine learning models (LASSO, Ridge, Elastic Net, random forest, KNN, and SVM) were applied to the 18 key interacting genes retained from the PPI network. Hyperparameter tuning confirmed that all models achieved stable performance under optimal settings; In the LASSO path, coefficients shrink to zero as log(λ) increases, with CXCL2, CSF1R, and ICAM1 persisting across a wide range, indicating their strong predictive roles. Ridge regression shows gradual coefficient shrinkage without elimination, consistent with L2 regularization. Elastic net (α = 0.5) exhibits intermediate behavior, combining feature selection with coefficient stability. For random forest, OOB error drops sharply and stabilizes after ~100 trees, confirming efficient convergence. KNN in PCA space reveals clear group separation. Importantly, SVM achieves complete discrimination, with all decision values for control and eCRSwNP samples falling on opposite sides of the threshold, highlighting its excellent classification power for distinguishing the two groups. (**Supplementary Figures 3A–F**). Feature selection demonstrated both cross-model convergence and algorithm-specific differences (**Supplementary Table 7**). LASSO retained HIF1A, RAC2, NCF2, and SELP, whereas Ridge preserved all 18 genes with relatively distributed coefficients (**Figures 5A, B**). Elastic Net also prioritized HIF1A, RAC2, NCF2, NCF4, and SELP, while retaining several correlated features such as TYROBP and HMOX1 (**Figure 5C**). Among the nonlinear models, random forest assigned the highest importance to NCF4, HIF1A, HMOX1, NCF2, and RAC2, whereas KNN and SVM primarily highlighted SELP, HIF1A, RAC2, and NCF2 (**Figures 5D–F**). To identify consistently prioritized features across models, we integrated the results across models using SHAP-based consistency and normalized importance analyses. SHAP consistency analysis showed that HIF1A, RAC2, and SELP clustered in the lower-left region, showing relatively consistent importance across different models; conversely, genes such as TYROBP, HMOX1, and SYK exhibited greater model dependence (**Figure 5G**).

The comparative analysis results of linear and nonlinear machine learning models indicate that HIF1A, RAC2, SELP, NCF2, and NCF4 all have relatively high feature importance in each algorithm (**Figures 5H, I**). After integrating and summarizing the standardized importance scores of all models, it was found that the combined scores of these five genes ranked the highest, with the scores being 6.453, 5.298, 4.347, 4.261, and 3.455 respectively (**Figure 5J**; **Supplementary Table 8**). The cross-algorithm feature importance heatmap further confirmed that these five genes form a significant core gene cluster with high expression (**Figure 5K**). Based on the above machine learning screening results, this study ultimately identified HIF1A, RAC2, SELP, NCF2, and NCF4 as candidate oxidative stress-related genes for subsequent experimental validation and functional investigation.

### 3.5 SHAP interpretation and ROC analysis verify the discriminatory potential of the core genes

**Figure 6:**
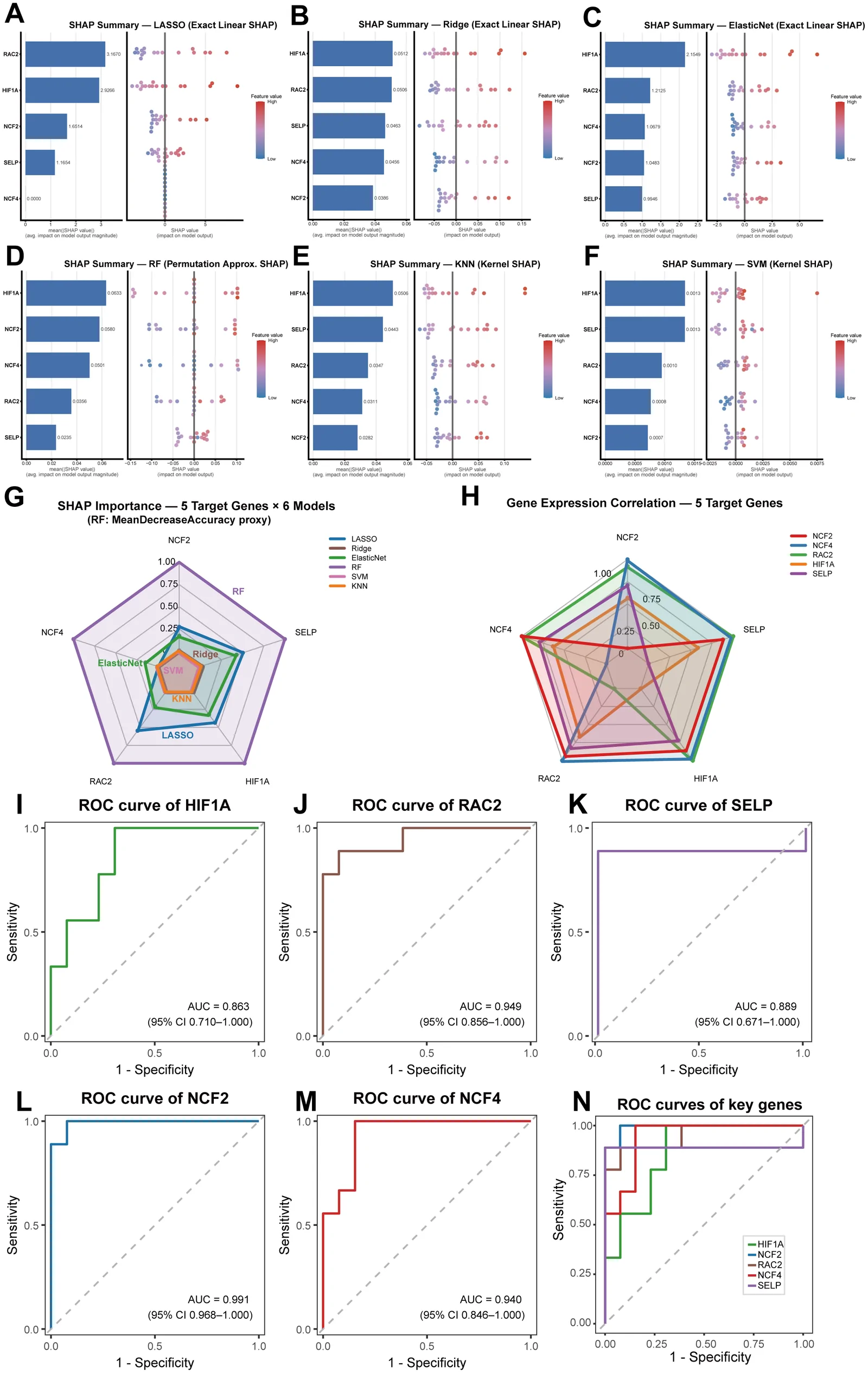
SHAP summary and ROC analyses of the five core genes. **(A–F)** SHAP summary plots in LASSO, Ridge, Elastic Net, random forest, KNN, and SVM models. Left plots show feature importance ranked by mean |SHAP|; right plots show SHAP beeswarm plots (dots represent samples, color indicates gene expression). **(G)** Radar plot of SHAP importance across six models. **(H)** Radar plot of absolute Pearson correlation coefficients among the five core genes. **(I–M)** ROC curves for HIF1A, RAC2, SELP, NCF2, and NCF4. **(N)** Overlay comparison of the five ROC curves.

To further analyze the predictive values of the five core genes, this study combined the SHAP algorithm to comprehensively analyze the feature contribution patterns of these genes in six machine learning models (**Figures 6A–F**). The results showed that although the rules for determining the feature weights of each algorithm differed, HIF1A, RAC2, SELP, NCF2, and NCF4 were all identified as high-value characteristic genes in multiple models. In linear models, HIF1A and RAC2 consistently ranked at the top of the influential features, and SELP and NCF2 also made significant contributions to the model determination; NCF4 was excluded in the LASSO model but retained in the Ridge and Elastic Net models, which is in line with the coefficient shrinkage principle of the sparse screening algorithm. In non-linear models, these genes still had good discriminatory value, but the feature rankings showed significant model heterogeneity: the random forest model focused on HIF1A and NCF2, while the KNN and SVM models used HIF1A and SELP as key features (**Figures 6A–F**). The radar chart visually confirmed the overall consistency of the analysis results of each machine learning model (**Figure 6G**). Gene correlation analysis indicated that there was a significant strong correlation between NCF2, NCF4, and RAC2, suggesting a close functional association among the three in the NADPH oxidase 2 (NOX2)-mediated oxidative stress axis (**Figure 6H**). The single-gene ROC diagnostic efficacy analysis demonstrated that all five core genes showed potential discriminatory ability between eCRSwNP and control samples, with area under the ROC curve (AUC) values of 0.863, 0.949, 0.889, 0.991, and 0.940 for HIF1A, RAC2, SELP, NCF2, and NCF4, respectively (**Figures 6I–N**; **Supplementary Table 9**).

**Figure 7:**
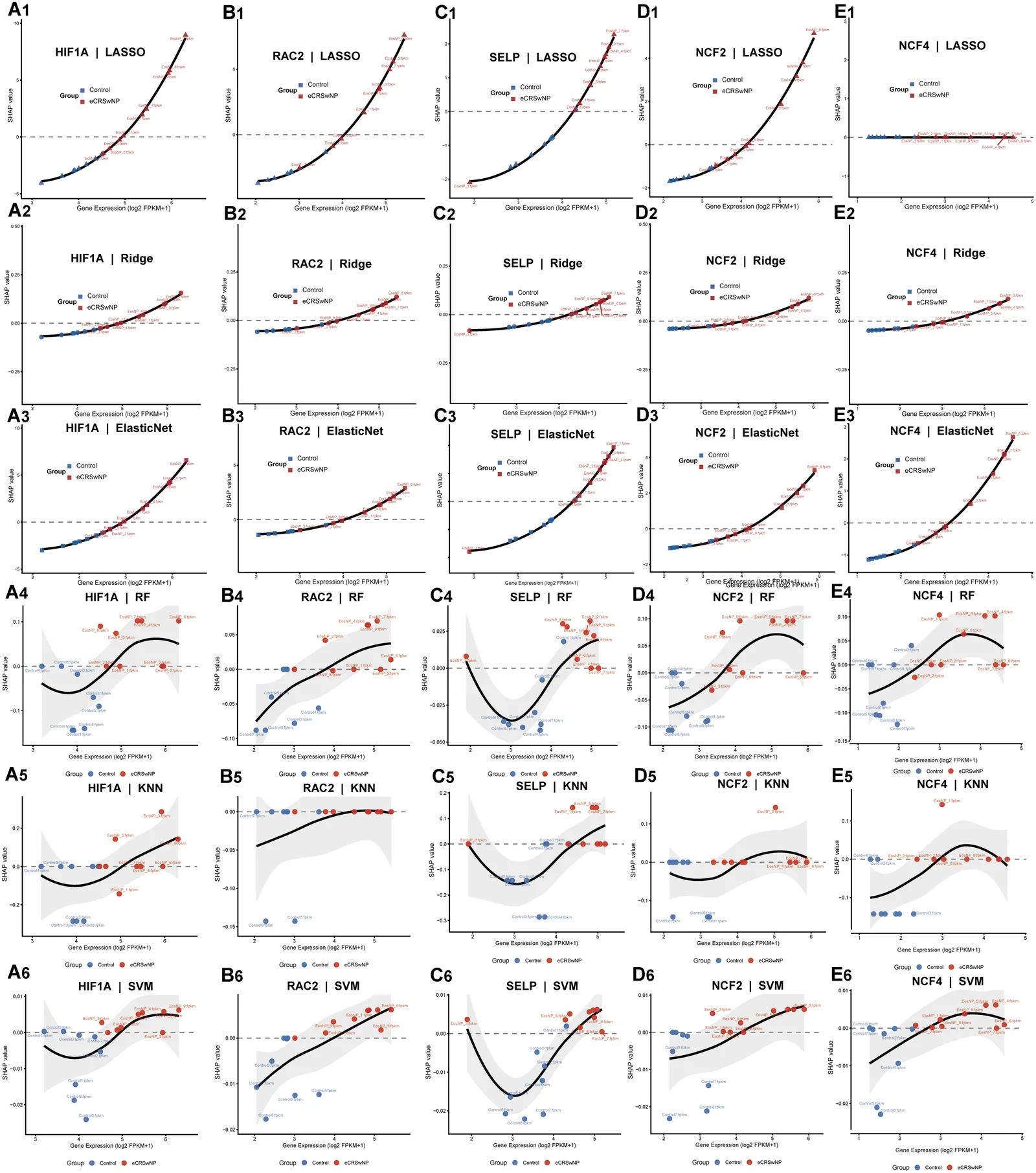
SHAP dependence plots of the five core genes across six machine-learning models. Plots for HIF1A **(A1–A6)**, RAC2 **(B1–B6)**, SELP **(C1–C6)**, NCF2 **(D1–D6)**, and NCF4 **(E1–E6)** across the respective models. Dots represent individual samples (blue: Control; red: eCRSwNP). The black curve indicates the fitted trend, the gray dashed line represents the SHAP = 0 reference, and gray shading denotes the uncertainty range in nonlinear models.

The SHAP dependence diagram can intuitively reflect the regulatory patterns of gene expression levels on the model prediction results (**Figure 7**). In linear models, the upregulation of HIF1A, RAC2, SELP, and NCF2 was usually accompanied by a steady increase in SHAP values, suggesting that these genes have a positive contribution to the determination of eCRSwNP disease; NCF4 had no significant contributing feature in the LASSO model, but showed a significant positive regulatory trend in the Ridge and Elastic Net models (**Figures 7A_1_–E_3_**). In non-linear models, the regulatory direction was basically the same as that in the linear model, but the mode of action was more complex. Among them, HIF1A and NCF2 showed a non-linear growth regulatory effect in random forest, KNN, and SVM models (**Figures 7A_4_-A_6_, D_4_–D_6_**); SELP and NCF4 showed a threshold-dependent or non-monotonic change pattern, indicating that the regulatory effect of these two genes on disease classification has conditional dependence (**Figures 7C_4_–C_6_, E_4_-E_6_**); RAC2 showed a stable positive regulatory trend overall, with differences in the strength of its effect in different non-linear models (**Figures 7B_4_–B_6_**).

### 3.6 Verification of the immune-specific expression characteristics of the core genes through single-cell transcriptome sequencing

**Figure 8:**
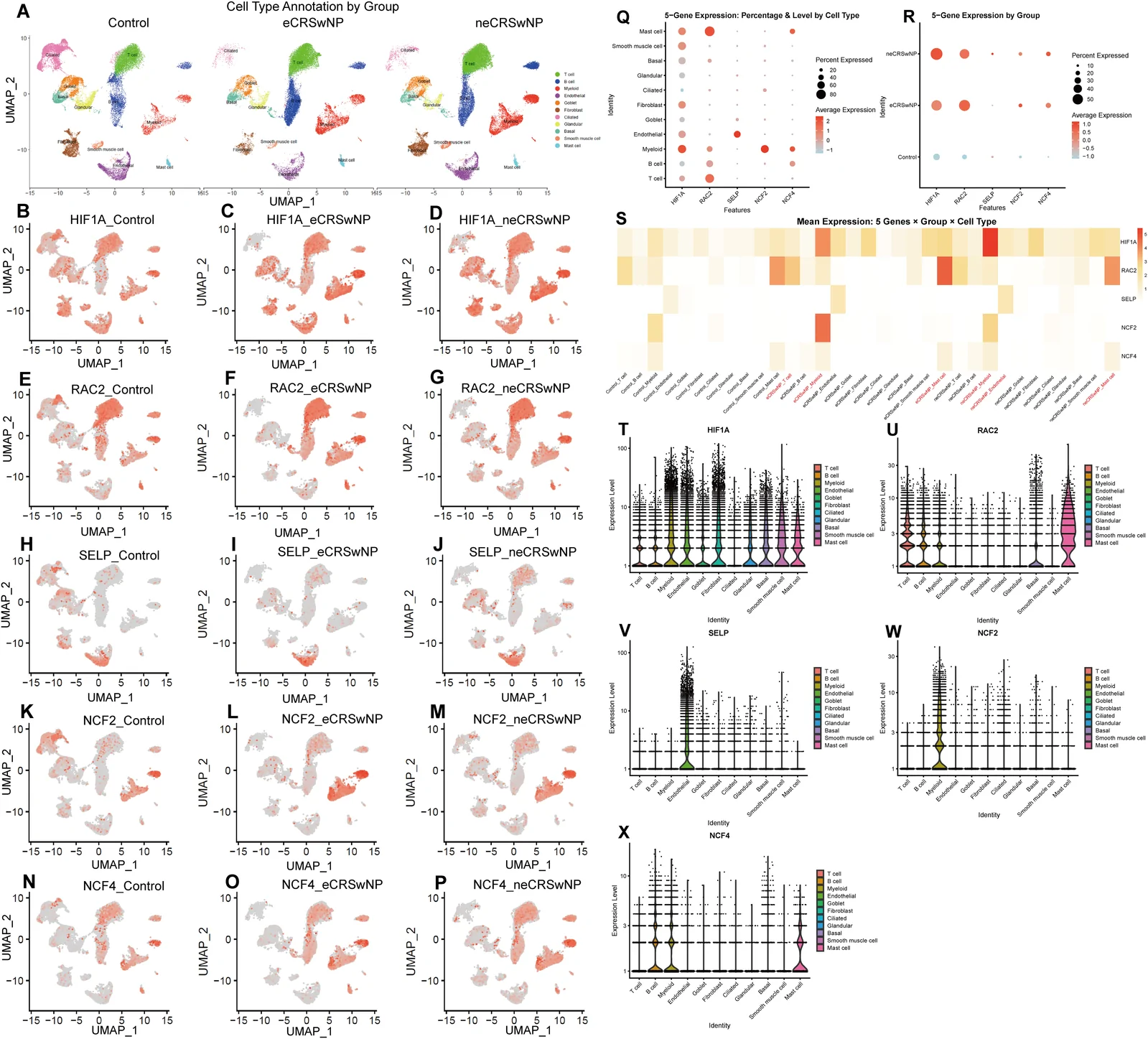
UMAP overview and cell-type distribution of five target genes in the nasal polyp scRNA-seq dataset. **(A)** UMAP plot with cell-type annotations. **(B–P)** Feature plots of HIF1A, RAC2, SELP, NCF2, and NCF4 across the three groups. **(Q)** Dot plot detailing the percentage of expressing cells and average expression across cell types. **(R)** Dot plot of expression patterns across groups. **(S)** Heatmap of mean expression across cell types and groups. The color scale gradient from green through yellow to red corresponds to mean gene expression values ranging from 1 to 5. **(T–X)** Violin plots mapping expression distributions across cell types.

To further analyze the cell distribution characteristics and expression patterns of the five core genes at the single-cell level, this study conducted verification analysis based on the publicly available single-cell transcriptome dataset of nasal polyps. This dataset included three clinical samples: normal control tissues, eCRSwNP, and neCRSwNP. UMAP dimensionality reduction analysis clearly divided the various cell subpopulations within the samples, and a total of 11 major cell groups were identified (**Figure 8A**). The gene feature distribution map confirmed that the five core genes exhibited significant cell type-specific expression (**Figures 8B–P**): HIF1A was widely expressed in various cells; RAC2, NCF2, and NCF4 were mainly enriched in myeloid cells; while SELP was specifically expressed in endothelial cells. The above expression distribution patterns were further verified by dot plots, cluster heatmaps, and violin plots (**Figures 8Q–X**). The comparison between groups showed that compared to the control group and the neCRSwNP group, the overall expression level of the core genes was higher in the eCRSwNP group samples, and the proportion of positive cells for the genes was significantly increased, with this difference being particularly evident in myeloid cells.

**Figure 9:**
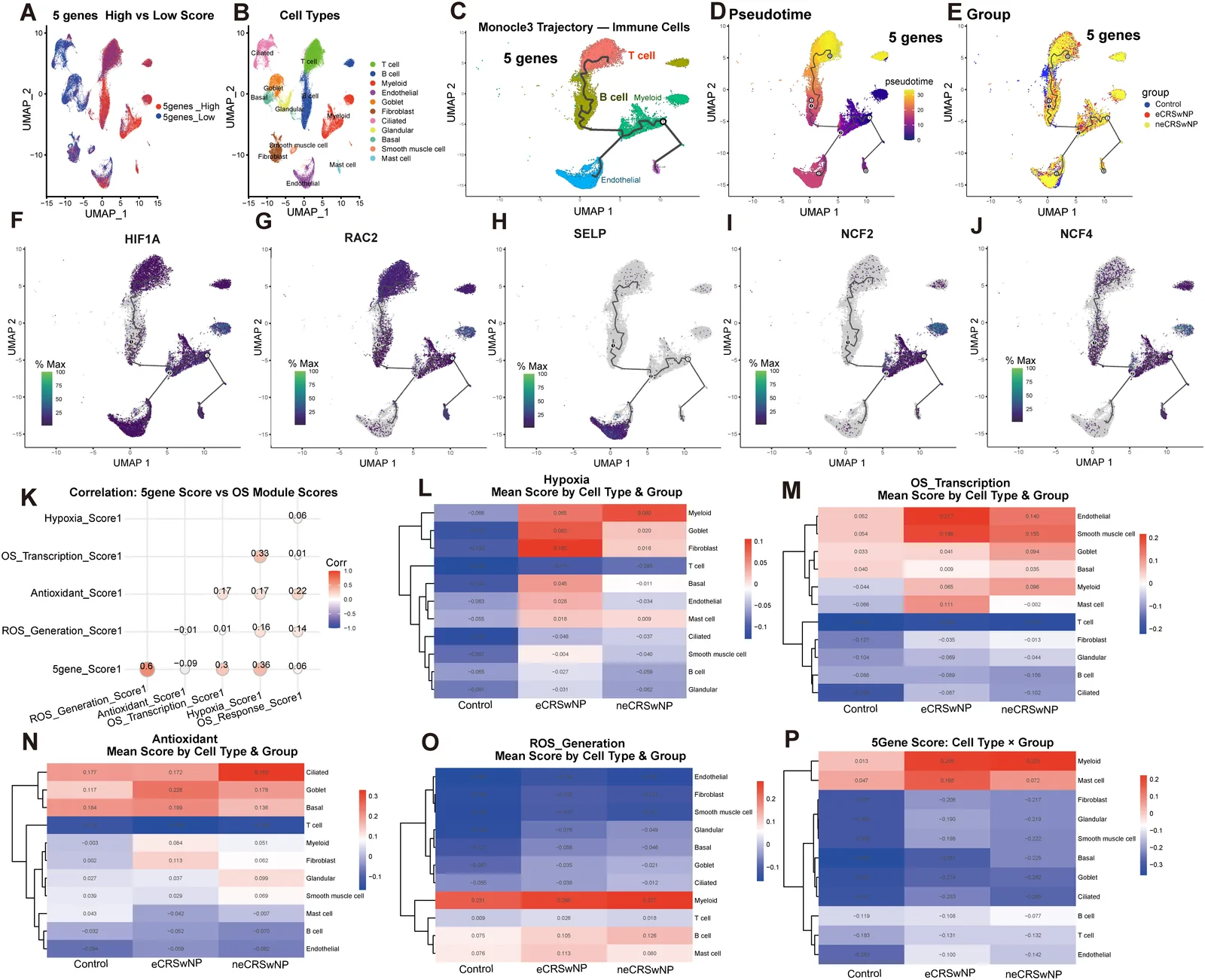
Distribution of the five-gene signature score and its association with oxidative stress–related module scores. **(A)** UMAP displaying the distribution of cells with high/low signature scores. **(B)** UMAP colored by cell type. **(C-E)** Monocle3 trajectory of immune cells colored by branch **(C)**, pseudotime **(D)**, and group **(E)**. **(F–J)** Feature plots of the five core genes mapped to the trajectory. **(K)** Correlation dot plot mapping associations between the signature score and oxidative stress modules. **(L–O)** Heatmaps of mean Hypoxia, OS_Transcription, Antioxidant, and ROS_Generation scores across cell types and groups. **(P)** Heatmap of the mean five-gene signature score across cell types and groups. The color scale gradient from blue through white to red denotes correlation coefficients and mean signature scores, which increase from low to high values.

This study constructed a five-gene characteristic scoring system based on single-cell expression profiles to quantitatively evaluate the cellular expression characteristics of gene combinations. The UMAP visualization results showed that cells with high five-gene characteristic scores mainly clustered in the immune cell distribution area, and were concentratedly enriched within the myeloid cell subgroups (**Figures 9A, B**). Using the Monocle3 algorithm for cell trajectory analysis, the immune cell population was mapped onto a continuous transcriptional pseudo-time axis, clarifying the temporal distribution characteristics of different immune subgroups (**Figures 9C–E**). The trajectory feature mapping results indicated that HIF1A, RAC2, NCF2, and NCF4 were highly expressed in the differentiation branches of immune cells, while the expression range of SELP was relatively limited (**Figures 9F–J**). Correlation analysis suggested that the five-gene characteristic score was most strongly associated with the ROS generation module, followed by the hypoxia pathway and the oxidative stress transcriptional regulatory module (**Figure 9K**). The corresponding clustering heatmap results confirmed that the ROS generation level in myeloid cells (especially those from eCRSwNP) was significantly increased in relation to the five-gene characteristic score (**Figures 9L–P**). In summary, the single-cell verification results indicated that this five-gene characteristic mainly participates in the activation process of immune cells, with myeloid cells as the main acting carriers, and is closely related to the oxidative stress regulatory pathway of eCRSwNP.

### 3.7 Nasal tissue sample experiments to verify the expression and localization characteristics of five core genes in CRSwNP

**Figure 10:**
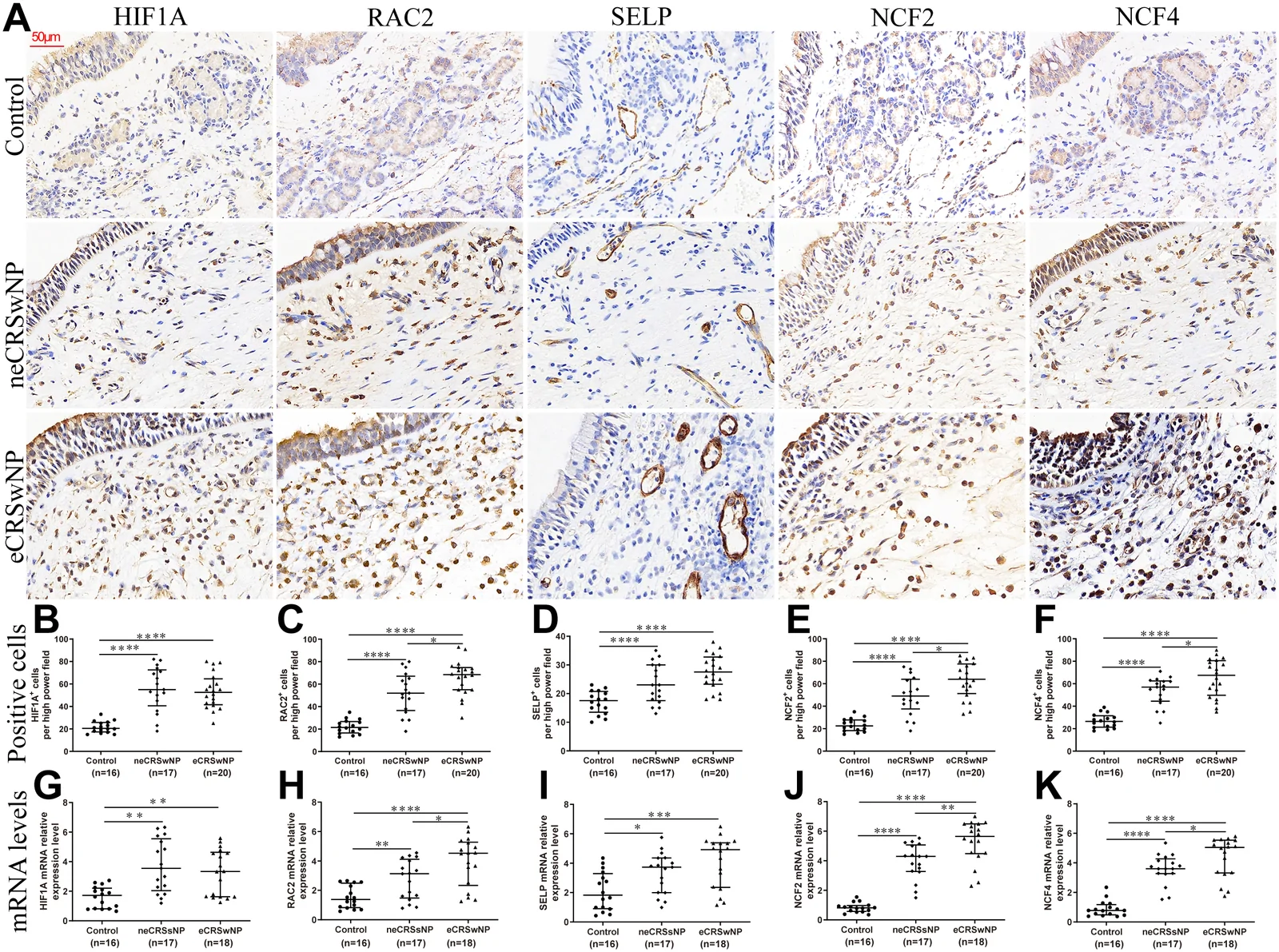
Expression and validation of key oxidative stress-related genes in different groups. **(A)** Representative immunohistochemical staining of the five core proteins (Scale bar = 50 μm). **(B–F)** Quantification of immunohistochemistry-positive cells. **(G–K)** Relative mRNA expression levels determined by qRT-PCR. *P < 0.05, **P < 0.01, ***P < 0.001, ****P < 0.0001.

To further validate the expression levels and spatial distribution characteristics of the core genes at the clinical tissue level, immunohistochemical staining was conducted in this study. The results showed that the positive cells expressing HIF1A, RAC2, SELP, NCF2, and NCF4 were relatively rare in normal nasal sinus mucosa tissues, but their positive cell expression was significantly upregulated in nasal polyp tissues. Among them, HIF1A was widely expressed in epithelial cells and interstitial inflammatory cells; RAC2, NCF2, and NCF4 were mainly enriched in the inflammatory cell infiltration areas, and the staining signals in eCRSwNP tissues were stronger; SELP was specifically enriched around the vascular structure (**Figure 10A**). The semi-quantitative statistical results indicated that the number of positive cells per high-power field of the five molecules in the polyp group was significantly higher than that in the control group; and the upregulation amplitude of RAC2, NCF2, and NCF4 positive cells in eCRSwNP was significantly higher than that in neCRSwNP (**Figures 10B–F**). The qRT-PCR transcriptional level detection results were consistent with the tissue staining results, and the expression of the five genes was all upregulated in nasal polyp tissues (**Figures 10G–K**).

**Figure 11:**
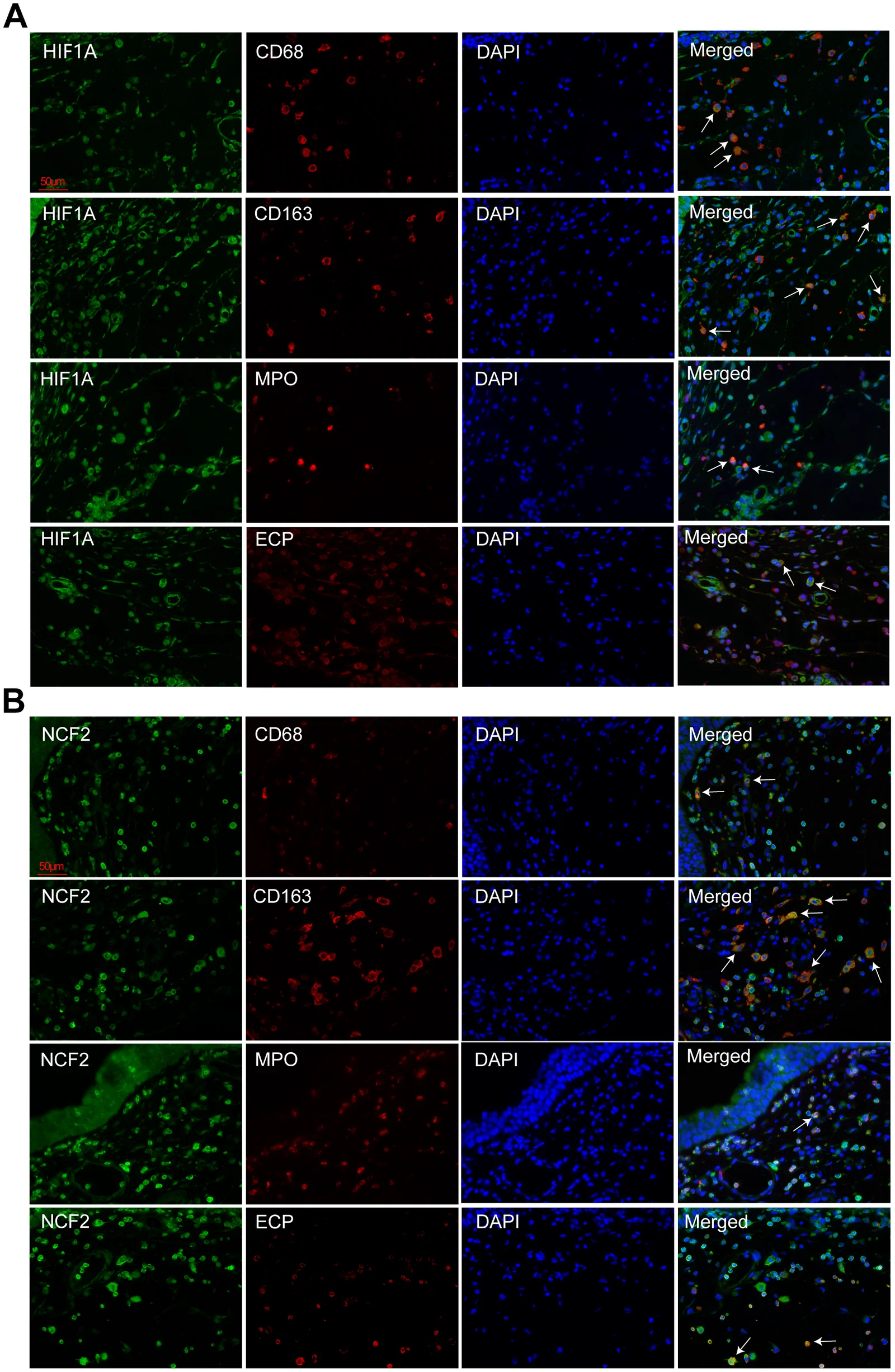
Immunofluorescence colocalization of HIF1A and NCF2 in nasal polyp tissues. Double immunofluorescence staining of HIF1A **(A)** and NCF2 **(B)** with CD68, CD163, MPO, and ECP. Panels (left to right) display the target protein (green), cell marker (red), DAPI-stained nuclei (blue), and merged image. Arrows indicate colocalization (Scale bar = 50 μm).

**Figure 12:**
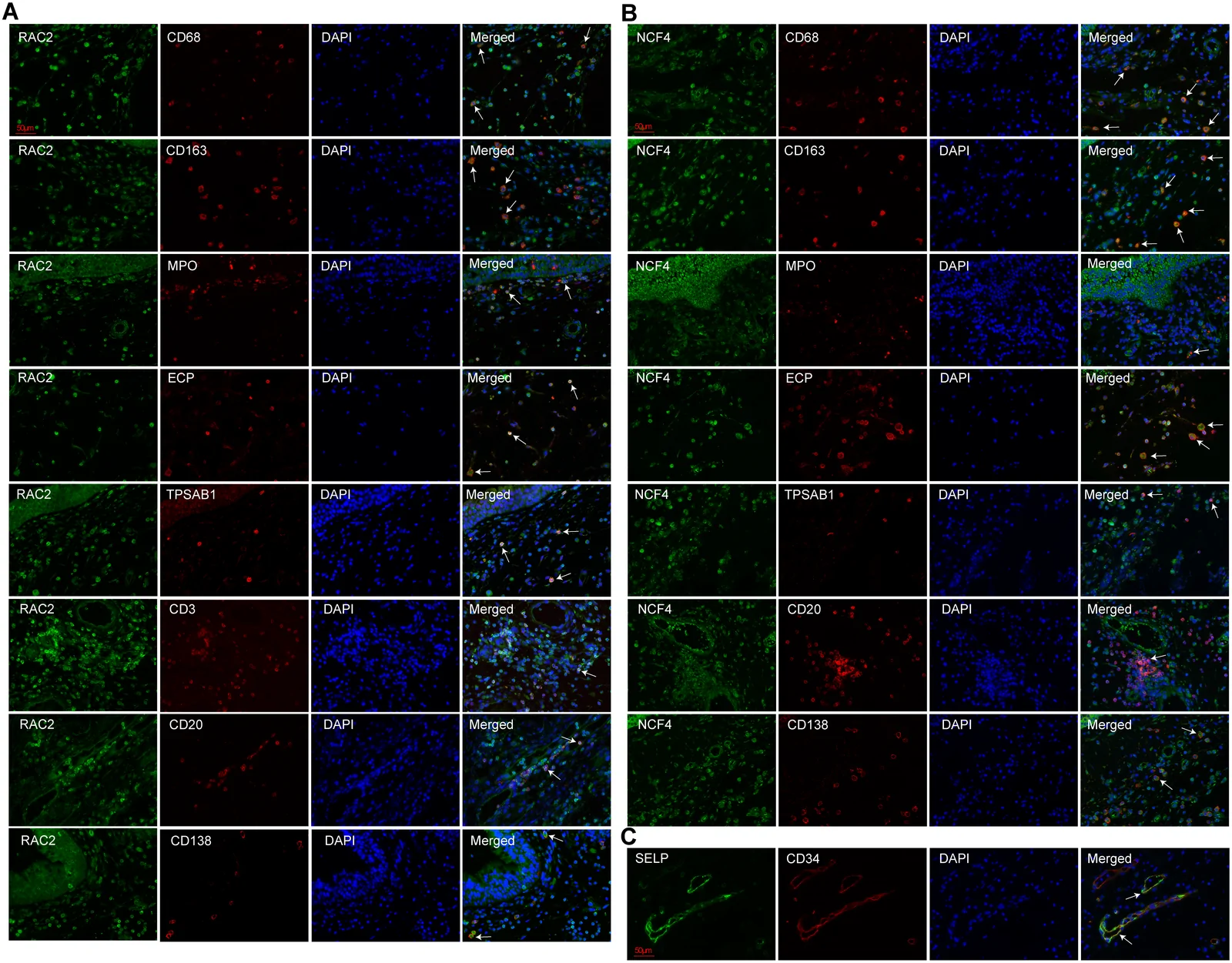
Immunofluorescence colocalization of RAC2, NCF4, and SELP in nasal polyp tissues. Double immunofluorescence staining of RAC2 **(A)**, NCF4 **(B)**, and SELP **(C)** with respective immune and endothelial markers. Panels (left to right) display the target protein (green), cell marker (red), DAPI-stained nuclei (blue), and merged image. Arrows indicate colocalization (Scale bar = 50 μm).

This study further employed immunofluorescence double-labeling staining to clearly determine the cellular localization characteristics of each molecule. Consistent with the results of single-cell sequencing, HIF1A could co-localize with pan-macrophage marker CD68, M2-type macrophage marker CD163, neutrophil marker MPO, and eosinophil marker ECP (**Figure 11A**); NCF2 also exhibited co-localization expression in the above immune cells (**Figure 11B**). The distribution range of RAC2 in immune cells was wider, and it could co-localize with macrophages, neutrophils, eosinophils, mast cells, T lymphocytes, B lymphocytes, and plasma cells markers (**Figure 12A**). NCF4 was mainly expressed in myeloid immune cells and co-localized with CD68, CD163, MPO, and ECP; positive signals could also be detected in mast cells, B cells, and plasma cells (**Figure 12B**). Unlike the above immune-related genes, SELP only specifically co-localized with the endothelial cell marker CD34, further confirming its endothelial cell-specific expression feature (**Figure 12C**).

## 4 Discussion

First, this study integrated multi-dimensional bioinformatics methods including transcriptome DEGs analysis, functional enrichment analysis, WGCNA, immune infiltration analysis, and PPI to confirm at the molecular level that CRSwNP is not a single inflammatory phenotype, but rather a chronic inflammatory syndrome with high molecular heterogeneity. Transcriptome DEGs analysis showed that eCRSwNP exhibited more significant transcriptome gene reprogramming changes compared to neCRSwNP. Simultaneously, comparative analysis of the three groups identified some common DEGs, suggesting that different CRSwNP endotypes and controls share a common inflammatory pathogenesis basis, and that each subtype forms a unique immune regulatory molecular network within this common inflammatory background.

Second, functional enrichment analysis further elucidated the differentiated immune-driving mechanisms of eCRSwNP and neCRSwNP. eCRSwNP is characterized by abnormal activation of biological processes such as cytokine interaction networks and immune cell chemotactic migration, consistent with the type 2 inflammation-dominated inflammatory cascade amplification pathological pattern; while neCRSwNP significantly enriched the IL-17 signaling pathway and innate immune activation pathway, suggesting that the maintenance of inflammatory homeostasis in this subtype mainly depends on type 3 inflammatory pathways. Existing studies indicate that the endotypes of CRSwNP are not completely independent, but rather exhibit a continuous spectrum distribution, with mixed endotypes coexisting (29, 30). Even in nasal polyp tissues where type 2 inflammation is dominant, different degrees of type 3 inflammation and neutrophil activation signals can still be detected (29). This crossover of inflammatory pathways is an important reason for the differences in clinical phenotype differentiation and drug treatment response among patients. The subtype molecular differences observed in this study do not negate the complexity of CRSwNP endotypes, but rather confirm that there are limitations to classifying subtypes solely based on clinical phenotypes. It is necessary to combine multi-dimensional molecular information such as gene network regulatory characteristics and mucosal immune microenvironment to improve the accuracy and interpretability of disease subtype stratification (31).

Third, this study conducted a systematic analysis with the oxidative stress-immuno-inflammatory interaction axis as the core entry point. The results confirmed that in eCRSwNP mucosal tissues, while immune-inflammatory pathways are abnormally activated, oxidative stress-related biological processes show a continuous activation trend. Current research on oxidative stress in CRSwNP has gradually shifted from the abnormal expression of a single biomarker to a deeper exploration of systemic redox imbalance in tissues, suggesting that oxidative stress may represent an important component of the inflammatory microenvironment and may be involved in the amplification of inflammatory responses in nasal polyps (32). The pathway enrichment analysis results of this study are consistent with the conclusions of existing studies. In addition to classic inflammatory pathways such as immune cell migration and inflammatory factor regulation, oxidative stress-related pathways involving ROS generation, particularly NADPH oxidase-associated superoxide production, were significantly enriched, suggesting a close association between oxidative stress-related signaling and immune-inflammatory pathways, which may contribute to inflammatory amplification in eCRSwNP (33).

Next, we applied six machine-learning models with different modeling strategies for candidate gene prioritization. By integrating standardized feature importance across multiple algorithms, we reduced the dependence on a single model and identified genes that showed relatively consistent contributions across different analytical approaches. SHAP-based interpretation further characterized the contribution patterns of these candidate genes in both linear and nonlinear models, providing additional insights into the relationship between gene expression patterns and model predictions. Based on this strategy, the overall ranking shows that HIF1A, RAC2, SELP, NCF2 and NCF4 maintain relatively consistent importance in different models and SHAP interpretations, suggesting that they have robust discriminative information across models; conversely, some genes only rank higher in specific nonlinear models and fall back in the overall ranking, suggesting that their contribution may depend on specific interaction structures or sample geometry, and their stability is relatively limited under small sample conditions. Overall, this integrative strategy provided a systematic approach for prioritizing candidate genes in this exploratory study (34).

NCF2 exhibited the highest single-gene discriminative power (AUC = 0.991) and consistently ranked high in multi-algorithm comprehensive feature ranking, suggesting strong and stable information content in distinguishing eCRSwNP from the control group. Combined with SHAP dependency analysis, the contribution of NCF2 showed a consistent positive change with increasing expression level; that is, the higher the NCF2 expression, the greater the model’s prediction probability for eCRSwNP, indicating a stable positive correlation between NCF2 and the eCRSwNP phenotype.

From a biological perspective, the elevated NCF2 in eCRSwNP tissues is more likely to reflect two complementary mechanisms. First, eCRSwNP is often accompanied by significant infiltration of myeloid immune cells and granulocytes, which are themselves the main expression and functional carriers of the NOX2 system; therefore, changes in cellular composition can directly increase NCF2 transcriptional signals at the tissue level (35). Secondly, in the chronic inflammatory microenvironment, the expression and activation of NOX2-related subunits can be regulated by multiple inflammatory signaling and metabolic stress networks (36). NOX2-derived ROS not only participate in host defense but can also act as redox signals to regulate pathways such as NF-κB, MAPK, and Nrf2, thereby affecting inflammation amplification and tissue homeostasis (37). The importance patterns of RAC2 and NCF2 are highly consistent in different algorithms, while NCF4 maintains a stable positive correlation with them, supporting that the NOX2-related pathway represented by RAC2–NCF2–NCF4 may represent an important oxidative stress-related molecular feature of eCRSwNP. SELP showed a certain discriminative ability in eCRSwNP (AUC = 0.889), suggesting that its increased expression can serve as a molecular clue for “local vascular endothelial activation and continuous recruitment of inflammatory cells”. A review of existing CRS mechanism studies has pointed out that P-selectin expression is enhanced in the vascular endothelium of nasal polyps, and blocking P-selectin can significantly inhibit the adhesion of eosinophils to the endothelium of nasal polyps, highlighting its role in the formation of eosinophilic inflammation in tissues (38).

HIF1A consistently ranks highly in the overall importance ranking of multivariate machine learning models, but its single-gene discriminative efficacy is relatively low (AUC = 0.863), suggesting that it is more likely to provide distinguishing information through synergistic effects with other features rather than as a highly specific single marker. Previous studies have shown that the inflammatory microenvironment of nasal polyps can exhibit a state of coexistence of local hypoxia and oxidative stress: hypoxia can induce the expression of epithelial-derived cytokines and promote the amplification of type II inflammation (39), while also promoting pathological processes related to epithelial-mesenchymal transition, thereby strengthening the coupling between inflammation and structural remodeling (40). On the other hand, ROS can promote HIF-1α stability under certain conditions by inhibiting PHD activity, so that hypoxic signals can be amplified even in non-hypoxic environments, and cross-link with inflammatory pathways (Redox regulation of HIF-1 signaling). Therefore, HIF1A expression may reflect the interaction between inflammatory burden, hypoxia-related responses, metabolic alterations, and tissue remodeling processes in eCRSwNP. The inclusion and stable contribution of HIF1A and NOX2-related genes (NCF2, RAC2, NCF4) in this study suggest that hypoxia/oxidative stress signals and the NOX2-ROS axis may be associated with inflammatory persistence and tissue remodeling in eCRSwNP. This also explains why multivariate models are better able to capture their discriminative value than single-gene analysis.

Subsequently, we validated the cellular origin of five key genes based on public nasal polyp scRNA-seq data, and further characterized their functional orientation in the inflammatory microenvironment using joint module scoring and pseudo-temporal analysis. NCF2, NCF4, and RAC2 showed the most concentrated expression enrichment in myeloid cells, SELP was mainly located in the endothelial cell region, while HIF1A was widely expressed in multiple cell types but was more prominent in inflammatory infiltration areas. Recent reviews have pointed out that single-cell technology is revealing significant heterogeneity between structural cells and immune cells and promoting a cellular-level understanding of the pathological mechanisms of CRS (38).

The five-gene joint score was highly clustered in the myeloid cell region on UMAP, and in intergroup comparisons, eCRSwNP showed a trend of being higher than neCRSwNP and the control, suggesting that the score is more likely to reflect “oxidative stress-related joint programs in the context of myeloid cell activation” rather than the random fluctuations of a single gene. The pseudo-chronological trajectory shows that myeloid cells are located in an earlier stage and branch out towards B cells and T cells. This result is not equivalent to the actual developmental lineage, but in the sense of the transition of inflammatory state, it suggests that myeloid cells may be in a more “initiation/hub” position, which is consistent with their key role in the initiation, amplification and interaction with structural cells of mucosal inflammation. The multiscale transcriptome study conducted by Liao et al. also emphasized the close coupling between immune-epithelial interaction and tissue remodeling, and regarded it as an important mechanistic framework for the persistence of chronic inflammation (22).

At the level of oxidative stress pathway association analysis, the five-gene joint score was most correlated with the ROS generation module score, and moderately correlated with the hypoxia and oxidative stress transcriptional regulation module, suggesting that NOX2-related oxidative burst and hypoxia/stress transcriptional program may jointly constitute key features of the myeloid microenvironment of eCRSwNP. Jiang et al. have proposed that NOX2-ROS can be coupled with inflammasome signaling and epithelial “alarm” related pathways, thereby promoting the persistence of mucosal inflammation (17).

Finally, we verified the upregulation trend of five key oxidative stress molecules in nasal polyp tissue at both the mRNA and positive expression cell levels using qRT-PCR and immunohistochemistry, and further clarified their tissue location and cell origin using immunofluorescence double staining. Overall, the upregulation at the mRNA level was consistent with the enhanced positive signal at the histological level, suggesting that the key genes screened earlier can be stably reproduced in real nasal polyp tissue. In particular, RAC2, NCF2, and NCF4 tended to show stronger expression enhancement in eCRSwNP, which is consistent with their functional positioning as key links in the activation and assembly of the NOX2 complex. It is worth mentioning that previous studies have observed upregulation of NCF2 expression in clinical eCRSwNP samples and its correlation with peripheral blood and tissue eosinophil indicators, suggesting that NCF2 can serve as one of the molecular clues reflecting eosinophilic inflammatory load and oxidative stress axis activation (41).

The colocalization results of IF have key value in the mechanism explanation: NCF2 colocalizes with myeloid cell markers (CD68, CD163) and granulocyte-related markers (MPO, ECP), RAC2 overlaps with myeloid cell, granulocyte and some mast cell markers, and NCF4 overlaps with B cell and plasma cell marker positive areas in addition to myeloid/granulocyte. SELP colocalizes linearly with CD34 positive vascular endothelial structures. These findings suggest two spatially distinct but potentially related histological patterns: NOX2-associated oxidative stress-related signals mainly enriched in immune-cell infiltration areas, and endothelial-associated adhesion signals located around vascular structures. Consistent with this observation, Zhou et al. reported that epithelial cells and macrophages are important cellular sources of oxidative stress signals in nasal polyps (32), while accumulating evidence suggests that redox imbalance and ROS-related pathways, including NADPH oxidase/NOX2-associated signaling, are closely associated with inflammatory activation in CRSwNP and other chronic inflammatory diseases (17, 20, 42). Together, these results suggest that, in addition to epithelial and macrophage-associated oxidative stress, the vascular–immune cell interface may also represent an important site associated with persistent inflammatory activation in eCRSwNP.

Several limitations of this study should be acknowledged. First, although multiple bioinformatic approaches, external single-cell transcriptomic validation, and tissue-level experimental verification were integrated, the relatively small cohort size represents an important limitation, particularly for machine-learning analyses and the small neCRSwNP subgroup. Given the heterogeneity of CRSwNP, the limited sample size may increase the risk of overfitting and restrict the statistical power and generalizability of the identified molecular signature. The five genes identified in this study should be considered candidate biomarkers rather than definitive diagnostic markers, and their clinical applicability requires further validation in larger, multicenter, and independent cohorts. Second, our machine learning algorithm only functioned as a feature selection tool without generating a trained classifier or complete predictive model. Thus, construction of a standardized large-sample predictive machine learning model is warranted for future investigations. Datasets would be split into a 70% training subset dedicated to gene screening and model training and a 30% internal test subset for independent ROC quantification, with rigorous segregation preserved for screening, training and validation datasets. Third, this was a single-center study and did not include clinical validation cohorts from other independent centers; therefore, the five genes identified in this study should be considered candidate biomarkers rather than definitive diagnostic markers, and their clinical applicability requires further validation in larger, multicenter, and independent cohorts. Fourth, although nasal polyp tissue represents the primary pathological compartment of CRSwNP, differences in anatomical origin between control inferior turbinate mucosa and nasal polyp tissue may introduce potential tissue-specific transcriptional variations. Finally, we merely validated the correlation between the five target genes and oxidative stress based on transcriptome profiling, bioinformatics mining, machine learning screening, cross-verification with public single-cell RNA sequencing databases, and experimental assays on clinical tissue specimens. However, thorough mechanistic research to clarify how these five genes mediate oxidative stress was not performed. Specifically, we lacked critical functional experiments: measuring tissue ROS levels via dihydroethidium (DHE) or 2′,7′-Dichlorofluorescein diacetate (DCFH-DA) staining, separately perturbing each gene in target cells or nasal polyp animal models, and monitoring subsequent ROS fluctuations in these two experimental systems. Further comprehensive investigations are required to unravel these underlying mechanisms in follow-up work.

## 5 Conclusion

Integrated multi-level analysis identified HIF1A, RAC2, SELP, NCF2, and NCF4 as key oxidative stress–related genes in eCRSwNP, with NCF2 showing the best diagnostic performance. Their validated upregulation and cell-type-specific distribution suggest that oxidative stress and inflammatory infiltration jointly contribute to eCRSwNP pathogenesis, providing candidate biomarkers and mechanistic insights for further investigation.

## Data Availability

The datasets presented in this study can be found in online repositories. The names of the repository/repositories and accession number(s) can be found below: https://www.ncbi.nlm.nih.gov/geo/, GSE338426.

## References

[1] Fokkens WJ Lund VJ Hopkins C Hellings PW Kern R Reitsma S . European position paper on rhinosinusitis and nasal polyps 2020. Rhinology. (2020) 58:1–464. doi: 10.4193/Rhin20.600 32077450

[2] Lialiaris ST Chaidas K Fyrmpas G Deftereou TE Spyroulia D Prokopakis EP . Inflammatory factors and chronic rhinosinusitis: an umbrella review. Cureus. (2026) 18:e101782. doi: 10.7759/cureus.101782 41710830 PMC12910526

[3] Lu RY Guo CL Liu Z . Stromal and immune cell interplay promotes neutrophilic inflammation in chronic rhinosinusitis with nasal polyps. Curr Opin Allergy Clin Immunol. (2026) 26:7–14. doi: 10.1097/ACI.0000000000001130 41451821

[4] Piazzetta GL Lobello N Di Agostino S Coscarella I Pelaia C Di Vito A . Mucosal remodeling in chronic rhinosinusitis with nasal polyps: the role of innate lymphoid cells and reprogramming under IL-4Ralpha blockade. Int J Mol Sci. (2026) 27:1992. doi: 10.3390/ijms27041992 41752128 PMC12941387

[5] Bachert C Hicks A Gane S Peters AT Gevaert P Nash S . The interleukin-4/interleukin-13 pathway in type 2 inflammation in chronic rhinosinusitis with nasal polyps. Front Immunol. (2024) 15:1356298. doi: 10.3389/fimmu.2024.1356298 38690264 PMC11059040

[6] Bachert C Marple B Schlosser RJ Hopkins C Schleimer RP Lambrecht BN . Adult chronic rhinosinusitis. Nat Rev Dis Primers. (2020) 6:86. doi: 10.1038/s41572-020-00218-1 33122665

[7] Wang X Zhang N Bo M Holtappels G Zheng M Lou H . Diversity of T(H) cytokine profiles in patients with chronic rhinosinusitis: a multicenter study in Europe, Asia, and Oceania. J Allergy Clin Immunol. (2016) 138:1344–53. doi: 10.1016/j.jaci.2016.05.041 27544740

[8] Wu AW Thapa M Alghezi M Oh E Perez HA Tang DM . The role of race and ethnicity in chronic rhinosinusitis with nasal polyps: a scoping review. Am J Rhinol Allergy. (2026) 40:81–90. doi: 10.1177/19458924251384391 41091890

[9] Wu AW Hur K Jafari A Ahmed OG Chen PG Takashima M . Histopathologic evaluation of Asian-American patients with chronic rhinosinusitis with nasal polyps. Int Forum Allergy Rhinol. (2024) 14:1822–6. doi: 10.1002/alr.23417 39046361

[10] Liao H Qiu C Luo Y Chen J Qin C Quan J . Safety and efficacy of anti-IL-5 monoclonal antibodies as second-line therapy for chronic rhinosinusitis with nasal polyps: a meta-analysis. Front Immunol. (2026) 17:1746573. doi: 10.3389/fimmu.2026.1746573 41983135 PMC13070943

[11] Wang J Li C Han J Xue Y Zheng X Wang R . Reassessing the roles of oxidative DNA base lesion 8-oxoGua and repair enzyme OGG1 in tumorigenesis. J BioMed Sci. (2025) 32:1. doi: 10.1186/s12929-024-01093-8 39741341 PMC11689541

[12] Manoharan RR Prasad A Pospisil P Kzhyshkowska J . ROS signaling in innate immunity via oxidative protein modifications. Front Immunol. (2024) 15:1359600. doi: 10.3389/fimmu.2024.1359600 38515749 PMC10954773

[13] Erysha Sabrina Jefferi N Afifah Shamhari A Abd Hamid Z Balkis Budin S Shima Taib I . Interlinkage between inflammation, oxidative stress, and endoplasmic reticulum stress in bisphenols-induced testicular steroidogenesis disturbance: a mini review. Int J Reprod BioMed. (2025) 23:17–32. doi: 10.18502/ijrm.v23i1.18187 40190456 PMC11966212

[14] Jomova K Raptova R Alomar SY Alwasel SH Nepovimova E Kuca K . Reactive oxygen species, toxicity, oxidative stress, and antioxidants: chronic diseases and aging. Arch Toxicol. (2023) 97:2499–574. doi: 10.1007/s00204-023-03562-9 37597078 PMC10475008

[15] Kracun D Lopes LR Cifuentes-Pagano E Pagano PJ . NADPH oxidases: redox regulation of cell homeostasis and disease. Physiol Rev. (2025) 105:1291–428. doi: 10.1152/physrev.00034.2023 39814410 PMC12285607

[16] Sies H Belousov VV Chandel NS Davies MJ Jones DP Mann GE . Defining roles of specific reactive oxygen species (ROS) in cell biology and physiology. Nat Rev Mol Cell Biol. (2022) 23:499–515. doi: 10.1038/s41580-022-00456-z 35190722

[17] Jiang S Zhang B Wen S Cheng S Shen Y Xie S . NOX2 mediates NLRP3/ROS facilitating nasal mucosal epithelial inflammation in chronic rhinosinusitis with nasal polyps. Heliyon. (2024) 10:e38029. doi: 10.1016/j.heliyon.2024.e38029 39328569 PMC11425172

[18] Huang ZQ Liu J Sun LY Ong HH Ye J Xu Y . Updated epithelial barrier dysfunction in chronic rhinosinusitis: targeting pathophysiology and treatment response of tight junctions. Allergy. (2024) 79:1146–65. doi: 10.1111/all.16064 38372149

[19] Meng L Liu S Luo J Tu Y Li T Li P . Oxidative stress and reactive oxygen species in otorhinolaryngological diseases: insights from pathophysiology to targeted antioxidant therapies. Redox Rep. (2025) 30:2458942. doi: 10.1080/13510002.2025.2458942 39894944 PMC11792148

[20] Tai J Shin JM Park J Han M Kim TH . Oxidative stress and antioxidants in chronic rhinosinusitis with nasal polyps. Antioxidants (Basel). (2023) 12:195. doi: 10.3390/antiox12010195 36671057 PMC9854928

[21] Peng Y Zi XX Tian TF Lee B Lum J Tang SA . Whole-transcriptome sequencing reveals heightened inflammation and defective host defence responses in chronic rhinosinusitis with nasal polyps. Eur Respir J. (2019) 54:1900732. doi: 10.1183/13993003.00732-2019 31439685

[22] Liao G Nakayama T Zhu B Lee IT Yeung J Yeo YY . Multi-scaled transcriptomics of chronically inflamed nasal epithelium reveals immune-epithelial dynamics and tissue remodeling in nasal polyp formation. Immunity. (2025) 58:2593–608:e6. doi: 10.1016/j.immuni.2025.08.009 40945518 PMC12857809

[23] Pan Y Wu L He S Wu J Wang T Zang H . Identification of hub genes and immune cell infiltration characteristics in chronic rhinosinusitis with nasal polyps: bioinformatics analysis and experimental validation. Front Mol Biosci. (2022) 9:843580. doi: 10.3389/fmolb.2022.843580 36060258 PMC9431028

[24] Guo Q Dong D Qiao X Huang S Zhao Y . Hub genes, diagnostic model, and predicted drugs related to ferroptosis in chronic rhinosinusitis with nasal polyps. Med (Baltimore). (2024) 103:e40624. doi: 10.1097/MD.0000000000040624 39612457 PMC11608670

[25] Zhou J Wang H Wang J Zhou F . Discovering biomarkers for chronic sinusitis with nasal polyps: a study integrating bioinformatics analysis and experimental validation of macrophage polarization and metabolism-related genes. Front Bioinform. (2025) 5:1613136. doi: 10.3389/fbinf.2025.1613136 41030501 PMC12477252

[26] Yu G Wang LG Han Y He QY . clusterProfiler: an R package for comparing biological themes among gene clusters. Omics. (2012) 16:284–7. doi: 10.1089/omi.2011.0118 22455463 PMC3339379

[27] Langfelder P Horvath S . WGCNA: an R package for weighted correlation network analysis. BMC Bioinf. (2008) 9:559. doi: 10.1186/1471-2105-9-559 19114008 PMC2631488

[28] Newman AM Liu CL Green MR Gentles AJ Feng W Xu Y . Robust enumeration of cell subsets from tissue expression profiles. Nat Methods. (2015) 12:453–7. doi: 10.1038/nmeth.3337 25822800 PMC4739640

[29] Oka A Klingler AI Kidoguchi M Poposki JA Suh LA Bai J . Effects of type 3 and neutrophilic inflammation on type 2 chronic rhinosinusitis with nasal polyps. J Allergy Clin Immunol. (2026) 157:374–86. doi: 10.1016/j.jaci.2025.09.023 41067280 PMC12594462

[30] Wang M Zhang N Zheng M Li Y Meng L Ruan Y . Cross-talk between T(H)2 and T(H)17 pathways in patients with chronic rhinosinusitis with nasal polyps. J Allergy Clin Immunol. (2019) 144:1254–64. doi: 10.1016/j.jaci.2019.06.023 31271788

[31] Toppila-Salmi S Reitsma S Hox V Gane S Eguiluz-Gracia I Shamji M . Endotyping in chronic rhinosinusitis-an EAACI task force report. Allergy. (2025) 80:132–47. doi: 10.1111/all.16418 39641584 PMC11724251

[32] Zhou J Zhou J Liu R Liu Y Meng J Wen Q . The oxidant-antioxidant imbalance was involved in the pathogenesis of chronic rhinosinusitis with nasal polyps. Front Immunol. (2024) 15:1380846. doi: 10.3389/fimmu.2024.1380846 38756779 PMC11096511

[33] Zhou D Fang D Yi J Li T Shi C Wu C . NADPH oxidase coordinating with ROS regulation: a biochemical footprinting in plant development. Physiol Plant. (2025) 177:e70668. doi: 10.1111/ppl.70668 41362101

[34] Javaid H Petrescu CC Schmunk LJ Monahan JM O'Reilly P Garg M . The impact of artificial intelligence on biomarker discovery. Emerg Top Life Sci. (2025) 8:89–105. doi: 10.1042/ETLS20243003 40924944 PMC12802349

[35] Zhong S Li Y Chen Y Jiang W Zheng J Wu L . Inhibition of ferroptosis-related NCF2 blocks the progression of lupus nephritis by activating PPARalpha pathway. Hereditas. (2025) 162:201. doi: 10.1186/s41065-025-00547-9 41035016 PMC12487152

[36] Wang Z Wu F Yan J Liang L Chang F Dong M . Ecdysterone alleviates atherosclerosis by inhibiting NCF2 and inhibiting ferroptosis mediated by the PI3K/Akt/Nrf2 pathway. J Cell Mol Med. (2025) 29:e70446. doi: 10.1111/jcmm.70446 40045169 PMC11882393

[37] Tocu G Stefanescu BI Stavar Matei L Tocu L . Phagocyte NADPH oxidase NOX2-derived reactive oxygen species in antimicrobial defense: mechanisms, regulation, and therapeutic potential-a narrative review. Antioxidants (Basel). (2025) 15:55. doi: 10.3390/antiox15010055 41596113 PMC12837977

[38] Li JX Wang ZC Liu Z Yao Y . Progress in cellular mechanisms of chronic rhinosinusitis. Fundam Res. (2026) 6:477–88. doi: 10.1016/j.fmre.2024.10.001 41647543 PMC12869774

[39] Zhang M Tang B Huang L Xiong Y Tu J Jia Y . Hypoxia induces the production of epithelial-derived cytokines in eosinophilic chronic rhinosinusitis with nasal polyps. Int Immunopharmacol. (2023) 121:110559. doi: 10.1016/j.intimp.2023.110559 37364325

[40] Pan S Zhuang M Wang X Zhang Q He T Li Y . Targeting PDK1: a novel approach to combat hypoxia-induced epithelial-mesenchymal transition in chronic rhinosinusitis with nasal polyps. Clin Transl Allergy. (2025) 15:e70048. doi: 10.1002/clt2.70048 40176272 PMC11964949

[41] Jia Y Nie J Wang H Han Z Zhang Z Li L . Correlation study of NCF2 in chronic rhinosinusitis with nasal polyps. Lin Chuang Er Bi Yan Hou Tou Jing Wai Ke Za Zhi. (2024) 38:303–9. doi: 10.13201/j.issn.2096-7993.2024.04.008 38563173 PMC11387289

[42] Tsai YJ Hsu YT Ma MC Wu CK Luo SD Wu WB . Transcriptomic analysis of genes associated with oxidative stress in chronic rhinosinusitis patients with nasal polyps: identifying novel genes involved in nasal polyposis. Antioxidants (Basel). (2022) 11:1899. doi: 10.3390/antiox11101899 36290622 PMC9598890

