# Supplementary material for: Integration of transcriptomics and machine learning to explore inflammatory characteristics and key oxidative stress–related molecules in nasal polyps
Source: Front Immunol. 2026 Aug 26;17:1879295. doi: 10.3389/fimmu.2026.1879295 (PMC13553475; doi:10.3389/fimmu.2026.1879295)
Supplement: Supplementary file 1 [file SupplementaryFile1.docx]

Supplementary Material

**Integration of transcriptomics and machine learning to explore inflammatory characteristics and key oxidative stress–related molecules in nasal polyps**

# 2 Methods

**2.1 Study design and workflow**

This study aimed to systematically integrate various screening strategies, including bulk transcriptome DEGs analysis, gene interaction regulatory network analysis, machine learning feature selection and screening, external validation using public single-cell sequencing data sets, and experimental verification using clinical nasal mucosa tissue samples. We conducted multi-level screening and systematic validation of key oxidative stress characteristic genes closely related to the inflammatory process of eCRSwNP. First, we collected nasal mucosa tissue samples from healthy control groups, neCRSwNP and eCRSwNP for RNA sequencing. Second, various bioinformatics analysis methods including DEGs screening, functional enrichment analysis, ingenuity pathway analysis (IPA), WGCNA, immune infiltration analysis, and protein-protein interaction (PPI) network analysis were performed sequently. Then, through the analysis of Venn intersection, the DEGs, genes involved in inflammatory regulation modules, and genes with characteristics of oxidative stress were cross-screened to initially obtain the key oxidative stress key genes highly related with eCRSwNP. To further eliminate redundant genes, prioritize candidate genes with higher discriminatory potential and improve feature selection efficiency, six different algorithm principles-based machine learning models were constructed to objectively score the importance and prioritize the selected characteristic genes. This further compressed and screened out high-identification core genes. Finally, based on the publicly available scRNA-seq data sets, the cell distribution and expression characteristics of the key genes were analyzed at the single-cell level, and tissue experiments were conducted using clinical nasal mucosa tissue samples to verify the expression patterns and distribution perspectives of the candidate genes.

**2.2 Patients and sample collection**

Patients who underwent functional endoscopic sinus surgery (FESS) or sinonasal surgery in the Department of Otolaryngology-Head and Neck Surgery between August 2023 and July 2024 were prospectively enrolled. The cohort included 22 subjects: 8 controls, 9 patients with eCRSwNP, and 5 patients with neCRSwNP. CRSwNP, eCRSwNP, and neCRSwNP were diagnosed according to the EPOS 2020 criteria based on clinical presentation, nasal endoscopy, sinus computed tomography, and histopathological evaluation. Ten non-overlapping high-power fields (HPFs) were randomly selected, and patients with an average eosinophil count ≥10/HPF were classified as eCRSwNP, whereas those with <10/HPF were classified as neCRSwNP. The inclusion criteria are listed below: (1) Participants were divided into two groups: adult patients with CRSwNP diagnosed per the EPOS 2020 diagnostic criteria, and control individuals free of chronic inflammatory sinonasal disorders; (2) no oral/nasal glucocorticoids, antibiotics, or biologics had been used by any participant within four weeks before surgery. Control nasal mucosa tissue samples were obtained from inferior turbinate mucosa of patients undergoing septoplasty for deviated nasal septum, whereas CRSwNP samples were collected from nasal polyp tissues during FESS. Patients with established antrochoanal polyps, immunodeficiency, coagulation disorder, cystic fibrosis, fungal sinusitis or a history of aspirin sensitivity were excluded. In addition, previous sinonasal surgery history has been recorded, and 4 patients with eCRSwNP and 1 patient with neCRSwNP underwent revision surgery, while the remaining patients had no history of previous nasal surgery. Detailed characteristics of enrolled subjects are listed in Supplementary Table 1.

Fresh tissue specimens were immediately rinsed with sterile saline to remove surface blood and secretions. The tissues were then divided into two parts: one part was placed in an RNase-free centrifuge tube, rapidly frozen in liquid nitrogen, and then transferred to −80°C for storage, to be used for subsequent RNA extraction; the other part was fixed in 4% paraformaldehyde fixative for paraffin embedding and hematoxylin and eosin staining (H&E). The fixed tissues were dehydrated, cleared, paraffin-embedded, and embedded sequentially to prepare 5 μm thick serial sections for H&E staining. Ten fields of view were randomly selected under a 400x microscope to count the number of eosinophils with typical morphological characteristics.This study was approved by the Ethical Committee of Shanghai Sixth People's Hospital Affiliated to Shanghai Jiao Tong University School of Medicine, and all subjects provided informed consent.

**2.3** **Bulk** **RNA sequencing and preprocessing**

Total RNA extracted from frozen tissues were used for bulk RNA sequencing. The concentration and purity of total RNA solution were determined using a NanoDrop-2000 spectrophotometer (Thermo Fisher Scientific, USA). Protein and organic solvent residues were assessed by measuring the OD260/280 and OD260/230 ratios. RNA integrity was measured using an Agilent 2100 Bioanalyzer (Agilent Technologies, USA), obtaining RNA integrity number (RIN) values. RNA integrity was comprehensively evaluated in conjunction with 28S and 18S rRNA peak shapes. Only samples meeting the requirements for high-throughput sequencing in terms of RNA purity and integrity were included for subsequent library construction and transcriptome sequencing analysis.

Quality-controlled RNA samples were sent to Shanghai Personalbio Technology Co., Ltd., for library construction and high-throughput sequencing. Library construction used Oligo(dT) magnetic beads to enrich mRNA with poly(A) tail structures from total RNA to reduce interference from rRNA and other non-coding RNAs. mRNA was randomly fragmented into small fragments of approximately 300 bp using ion fragmentation. The choice of fragment length was based on the technical characteristic of fixed-length sequencing adapters: fragments that were too short would increase the proportion of adapter sequences, thus reducing the proportion of effective sequencing data, while fragments that were too long would hinder cluster formation and signal acquisition during sequencing. Using the fragmented RNA as a template, first-strand cDNA was synthesized with reverse transcriptase and six-base random primers. Subsequently, second-strand cDNA was synthesized using the first-strand cDNA as a template, forming double-stranded cDNA. The double-stranded cDNA was amplified by polymerase chain reaction (PCR) for library fragment enrichment, and then screened according to fragment size, ultimately obtaining a sequencing library with an insert fragment length of approximately 450 bp.

After library construction, the Agilent 2100 Bioanalyzer (Agilent Technologies, USA) was used to perform quality checks on the fragment length distribution and integrity of the libraries, and to quantitatively analyze the total and effective concentrations of the libraries. Based on the effective concentrations of each library and the expected sequencing data volume, libraries with different index sequences were mixed proportionally. The mixed libraries were then uniformly diluted to 2 nM and subjected to alkaline denaturation to form single-stranded libraries. Finally, next-generation sequencing (NGS) technology was used on the Illumina sequencing platform to perform paired-end (PE150) sequencing of the libraries, obtaining raw sequencing data for subsequent transcriptome analysis.

Raw RNA-seq reads were subjected to quality assessment and preprocessing prior to gene quantification. Briefly, sequencing quality was evaluated using FastQC, followed by the removal of adapter sequences, low-quality bases, and short contaminating reads using Trimmomatic. The resulting high-quality clean reads were aligned to the human reference genome (GRCh38; Homo_sapiens.GRCh38.dna.primary_assembly.fa) using HISAT2. Gene-level read counts were subsequently quantified with featureCounts and used as the input for downstream differential expression analysis.

Raw counts were used for differential expression analysis, whereas fragments per kilobase of transcript per million mapped reads (FPKM) values were used for visualization, WGCNA, and immune infiltration analysis. Differential expression analysis was conducted using the DESeq2 package based on raw integer read counts. Following the standard DESeq2 workflow, normalization was performed using the median-of-ratios method, in which sample-specific size factors were estimated to account for differences in sequencing depth and library composition across samples. Dispersion parameters were then estimated for each gene using an empirical Bayes approach, and gene expression was modeled using negative binomial generalized linear models.

Principal component analysis (PCA) was performed after variance-stabilizing transformation to assess global transcriptomic differences among groups.

**2.4 DEGs and functional enrichment analyses**

DEGs were identified using the DESeq2 R package. Three pairwise comparisons were performed: control versus eCRSwNP, control versus neCRSwNP, and neCRSwNP versus eCRSwNP. In each comparison, the first group served as the reference group; therefore, a positive log2 fold change indicated higher expression in the second group, whereas a negative log2 fold change indicated lower expression in the second group. Genes with an adjusted *P* value < 0.05 and |log2 fold change| >= 1 were considered differentially expressed. Functional interpretation of DEGs was performed using Gene Ontology (GO), Kyoto Encyclopedia of Genes and Genomes (KEGG), and Gene Set Enrichment Analysis (GSEA). IPA was further used to predict canonical pathways and disease and function activation states.

DEGs analysis of raw counts was performed using the DESeq2 package (v1.38.3) in R (v4.5.1). DESeq2 uses a negative binomial distribution model to model the read count data and normalizes the sequencing depth differences between samples. The expression changes of genes in different groups are evaluated by estimating the dispersion parameter. In this study, pairwise comparisons were made between the eCRSwNP, neCRSwNP and Control groups.

GO, KEGG, and GSEA analyses utilized the "clusterProfiler" R package (v4.18.4). The gene list was pre-ranked by fold changes for GSEA, using canonical pathways gene sets from the KEGG pathway database (mSigDB C2 category) downloaded from the "Molecular Signatures Database v2023.1."

The results of DEGs containing gene identifiers, corresponding log2 fold changes, and *P* values were uploaded into the IPA software (Qiagen Bioinformatics, Redwood City, CA, USA; <https://digitalinsights.qiagen.com/>) and analyzed with the "core analysis" function. Enrichment pathways of DEGs were generated based on the Ingenuity Pathway Knowledge Data Base. The results of enriched pathways were ranked by -log (*p*-value).

**2.5 Co-expression network analysis and immune infiltration analysis**

WGCNA analysis was performed using the WGCNA package (v1.73) in R (v4.5.1). After filtering low-expression and low-variation genes in the FPKM expression matrix, hierarchical clustering and network connectivity analysis were performed on the samples to assess the overall expression similarity between samples and identify potential abnormal samples. Based on the principle of scale-free network construction, the changing trends of the scale-free topology fit index (R²) and mean connectivity of the network under different soft threshold parameters β were evaluated. The minimum β value that makes R² reach 0.8–0.9 for the first time and the mean connectivity remain at a relatively reasonable level was selected as the optimal soft threshold using the pickSoft Threshold function. A weighted adjacency matrix was constructed based on this soft threshold parameter, and the topological overlap matrix (TOM) was further calculated to measure the topological similarity between genes, thereby comprehensively reflecting the direct and indirect co-expression relationship between genes. The genes were hierarchically clustered using TOM dissimilarity as a distance metric, a gene clustering tree was constructed, and a dynamic tree cut algorithm was combined to identify co-expression modules. Different modules are identified by different colors, with the "grey" module containing all genes not assigned to any specific module. A module eigengene (ME) value is calculated for each module as a representative of its overall expression pattern. Pearson correlation analysis is used to calculate the correlation coefficient between the ME values of each module and clinical characteristics, along with their corresponding p-values; *p* < 0.05 is considered statistically significant. A module-trait correlation heatmap is constructed to identify key modules that are significantly positively correlated with eCRSwNP. To identify gene modules associated with eosinophilic inflammation, WGCNA was performed on the filtered expression matrix. Module eigengenes were correlated with clinical eosinophilic traits, including blood eosinophil percentage, blood eosinophil count, tissue eosinophil percentage, and tissue eosinophil count. Modules significantly associated with the eosinophilic endotype were selected for downstream analysis.

In R, the CIBERSORT (Cell-type Identification By Estimating Relative Subsets Of RNA Transcripts) algorithm v1.04 was used for immune cell infiltration analysis. CIBERSORT is a deconvolution algorithm based on support vector regression (SVR). It infers the relative abundance of each immune cell subset by fitting the gene expression profile of mixed tissue samples to a predefined immune cell feature gene expression matrix. The input data was the standardized FPKM expression matrix. The running parameters were kept at the default settings, and the number of permutations was set to 1000. The reference matrix was the LM22 feature gene set provided by CIBERSORT. This gene set contains the marker gene expression features of 22 human immune cell subsets, covering various T cell subsets (such as CD4⁺ memory T cells, CD8⁺ T cells, etc.), B cells, plasma cells, NK cells, macrophage subtypes (M0, M1, M2), as well as neutrophils, eosinophils, and mast cells. For each sample, CIBERSORT outputs the relative proportions of immune cell subsets and the overall fit significance *P*-value, with *P* < 0.05 considered statistically significant. The top 10 most abundant cell subsets were selected for subsequent visualization and inter-group difference comparisons. Stacked bar charts were used to display the overall composition proportions of these immune cell subsets in each group of samples, violin plots combined with scatter plots were used to show their distribution characteristics across different groups, and Kruskal–Wallis tests were used to compare differences among the three groups. Dunn post-hoc tests were used for pairwise comparisons, and the Benjamini–Hochberg method was used to correct for multiple test errors.

Furthermore, Pearson correlation analysis was used to calculate the correlation coefficients and corresponding *P*-values between the proportions of each immune cell subset and the module ME values obtained from WGCNA analysis, and a correlation heatmap was constructed for visualization, revealing the association characteristics between immune cell infiltration patterns and key co-expressed modules at the system level.

**2.6 Candidate oxidative stress-related gene identification and PPI analysis**

Oxidative stress-related genes were obtained by searching the GeneCards database using the keyword "oxidative stress". To improve the reliability of the candidate gene set, only genes with a Relevance Score ≥ 7 were included as the oxidative stress-related gene set. Based on this, the intersection of the selected DEGs, genes in the WGCNA analysis module significantly associated with the eCRSwNP endotype, and the oxidative stress-related gene set was taken to obtain the candidate oxidative stress-related gene set for subsequent analysis.

PPI network analysis was used to further analyze the functional associations among candidate oxidative stress-related genes and their central role in the molecular network from the perspective of protein-protein interaction. The selected candidate genes were imported into the STRING database (search tool for the retrieval of interacting genes/proteins, https://string-db.org/) for interaction analysis, with the species limited to Homo sapiens. The combined score of the interaction confidence was set to ≥0.4 to screen for protein-protein interaction relationships with reliable supporting evidence. The network construction comprehensively considered evidence from multiple sources, including experimental verification, database annotation, co-expression, and text mining. The obtained PPI network results were imported into Cytoscape software for visualization, and the CytoHubba plugin was used to perform topological analysis on the network to identify key nodes with potential core regulatory roles in the network. All nodes were sorted based on the node degree algorithm, and the genes with the top 50% connectivity were selected as core network genes for further screening by subsequent machine learning algorithms.

**2.7 Machine-learning analysis and feature selection**

Considering the small sample size of neCRSwNP (n=5), including it in multi-class machine learning modeling would significantly increase model complexity and the risk of overfitting. Therefore, this study focused its machine learning analysis on the binary classification problem between eCRSwNP and Control. A total of 17 samples were ultimately included.

Candidate feature genes were derived from DEGs in the previous Control vs eCRSwNP group, genes highly associated with eosinophilic inflammation in WGCNA, oxidative stress-related genes in the GeneCards database, and PPI network topology screening results. After intersection integration and PPI network screening, 18 core candidate genes were finally identified as machine learning input features: NCF2, NCF4, RAC2, HIF1A, SELP, TYROBP, PTPRC, ICAM1, HMOX1, CCL2, CXCR4, ITGB2, VCAM1, SYK, CYBB, CSF1R, SELL, and PECAM1.

**2.7.1Six Machine Learning Algorithm Modeling Frameworks**

**2.7.1.1Data Segmentation and Validation Strategies**

Given the small sample size (n=17), this study employs Leave-One-Out Cross-Validation (LOOCCV) as the primary model evaluation framework to maximize the utilization of training data while avoiding information leakage. Hyperparameter tuning is performed within the inner loop of LOOCV, ensuring that information from test samples does not infiltrate the hyperparameter selection process, thus obtaining unbiased generalization performance estimates.

**2.7.1.2 LASSO Regression (α=1, Pure L1 Regularization)**

LASSO regression achieves variable selection by adding an L1 penalty term (λΣ|βⱼ|) to the loss function: as λ increases, the regression coefficients of some features are precisely compressed to zero, thereby achieving automatic feature sparsity. This study implements LASSO regression using the glmnet package in R. Feature importance is measured by the absolute value of the standardized coefficients at the optimal λ.

**2.7.1.3 Ridge Regression (α=0, Pure L2 Regularization)**

Ridge regression suppresses coefficient inflation through an L2 penalty term (λΣβⱼ²) without producing sparse solutions—all 18 features retain non-zero coefficients. This property allows ridge regression to stably estimate coefficients in gene expression data with multicollinearity, especially suitable for scenarios where functionally related genes are highly correlated.

**2.7.1.4 ElasticNet (α=0.5)**

ElasticNet combines L1 and L2 penalty terms: Loss function = RSS + λ[α×Σ|βⱼ| + (1-α)/2×Σβⱼ²], where α is a mixing parameter (α=1 is equivalent to LASSO, α=0 is equivalent to Ridge). In gene expression data, when multiple related genes contribute to a biological pathway, resilient networks tend to include or exclude the entire group, while non-LASSO networks retain only one representative gene within the group, thus offering higher biological interpretability.

**2.7.1.5 Random Forest (RF)**

RF constructs 500 independent decision trees (each trained on bootstrap sampled data, with mtry features randomly selected at each node for splitting), and finally integrates the classification results using a voting mechanism. The introduction of randomness (including double randomization of samples and features) gives RF natural robustness against overfitting and resistance to noisy features. This study implemented it using the randomForest package in R (ntree=500), with an optimal mtry=2. Feature importance is assessed using the Mean Decrease Accuracy (MDA) metric based on permutations to ensure fair evaluation of relevant features. The out-of-bag (OOB) error curve of RF shows that it stabilizes after approximately 50 trees, indicating that 500 trees have converged sufficiently.

**2.7.1.6 K-Nearest Neighbors (KNN) Algorithm**

The KNN algorithm uses Euclidean distance as a metric, classifying samples by majority vote among their k nearest neighbors. Visualization in the principal component space (taking the top two PCs) shows good linear separability between the Control and eCRSwNP groups on the PC1 axis. Some eCRSwNP samples are distributed across different regions of PC1 due to their highly heterogeneous expression profiles, consistent with the clinical heterogeneity of eCRSwNP. Feature importance is calculated using a permutation method: the expression value of each gene is randomly shuffled, and the average decrease in predicted probability is used as the importance score for that gene.

**2.7.1.7 Support Vector Machine (SVM)**

SVM achieves high-dimensional data classification by finding a hyperplane that maximizes the classification margin. It uses radial basis function (RBF) kernels to map features to a high-dimensional space to handle non-linearly separable data. The hyperparameter grid search is jointly optimized by LOOCV, and the optimal parameters are C=0.1 and σ=1.

**2.7.2 Feature Importance Standardization and Multi-Algorithm Consensus Ranking**

To ensure fair comparison among the six algorithms, this study performs Min-Max standardization on the raw feature importance scores output by each algorithm (absolute coefficient values for linear models, MDA for RF, and permutation importance for KNN and SVM), mapping the score of each algorithm to the [0, 1] interval: Iₙₒᵣₘ = (I − Iₘᵢₙ) / (Iₘₐₓ − Iₘᵢₙ). After standardization, the gene with the highest importance in each algorithm receives a score of 1.0, and the gene with the lowest importance receives a score of 0.0.

The overall ranking is calculated based on the sum (or mean) of the standardized importance scores of each gene across the six algorithms, sorted in descending order of the sum to obtain the total aggregate ranking. Furthermore, the SHAP method is introduced as a seventh evaluation dimension, and the scores are standardized equally based on the SHAP mean |φ|, making the feature selection framework more comprehensive.

**2.7.3 SHAP Value Calculation and Interpretability Analysis**

For the three linear models (LASSO, Ridge, and ElasticNet), the SHAP value is calculated using the exact linear SHAP formula: φᵢ(x) = βᵢ × (xᵢ − E[xᵢ]), where βᵢ is the model coefficient under optimal λ, xᵢ is the gene expression value of the sample, and E[xᵢ] is the mean of genes in the training set. This formula is completely equivalent to the game-theoretic Shapley value under linear model conditions, requiring no Monte Carlo approximation or kernel function estimation, and has the highest computational accuracy.

For RF, a permutation-based approximation SHAP (i.e., permutation importance proxy) is used; for KNN and SVM, the Kernel SHAP method is used, approximating the SHAP value on the local hyperplane through weighted linear regression.

**2.7.4 Visualization Analysis**

This study adopted the following five SHAP visualization schemes: (1) SHAP Beeswarm Plot: The horizontal axis is the SHAP value and the vertical axis is the gene. Each point represents a sample, and the color code corresponds to the expression level of the gene (high expression = red, low expression = blue); (2) SHAP Feature Importance Bar Chart: The global contribution ranking of each gene is measured by the average |SHAP| value; (3) SHAP Dependence Plot: The scatter plot shows the relationship between the expression level of a specific gene (log₂ FPKM+1, horizontal axis) and the SHAP value (vertical axis). The color distinguishes between Control (blue) and eCRSwNP (red) samples, and a loss trend line is added; (4) Radar Chart: Shows the relative distribution of SHAP importance of 5 core genes in 6 models; (5) Cross-model SHAP Consistency Scatter Plot: The stability of each gene in the six models is evaluated by using the Mean SHAP ranking as the horizontal axis and the standard deviation of the SHAP ranking as the vertical axis.

**2.7.5 Single-gene ROC analysis and evaluation**

The pROC (v1.18.5) package in R language was used to perform single-gene ROC analysis and plot ROC curves for the five core feature genes, and the AUC and 95% confidence intervals were calculated (DeLong method). The comprehensive performance indicators of the model include: AUC-ROC, 95% confidence interval, sensitivity, specificity, accuracy, and confusion matrix.

**2.8 External scRNA-seq validation**

**2.8.1 Single-Cell RNA Sequencing Datasets and Preprocessing**

The nasal polyp scRNA-seq dataset used in this study came from the Genome Sequence Archive (GSA, HRA000772), containing the Control group (n=36,291 cells), the eCRSwNP group (n=28,500 cells), and the neCRSwNP group (n=39,751 cells), totaling approximately 104,000 cells. The dataset contained 22 nasal mucosal tissue samples, including 5 control nasal mucosal tissues and 11 nasal polyps (6 eCRSwNP and 5 neCRSwNP). The data underwent Harmony batch correction and integration, and has been standardized, screened for highly variable genes, PCA analysis, and UMAP dimensionality reduction clustering. Cell type annotations cover 11 cell types, including epithelial cells, basal cells, ciliated cells, goblet cells, fibroblasts, endothelial cells, smooth muscle cells, mast cells, myeloid cells, B cells, and T cells. All analyses were performed in the R language environment (version 4.5.1), using R Studio (version 2025.09.1), primarily based on the Seurat (v5.3.0), ggplot2 (v4.0.2), and Monocle3 (v1.3.4) packages.

**2.8.2 Basic Expression Panoramic Analysis**

The spatial distribution of five genes—HIF1A, RAC2, SELP, NCF2, and NCF4—in all cells was displayed using UMAP feature plots, grouped into three groups (Control/eCRSwNP/neCRSwNP). Violin plots (VlnPlot) were used to compare the expression differences of the five genes across different cell types and groups. Bubble plots (DotPlot) simultaneously displayed gene expression levels and positivity rates, and heatmaps presented the average expression values of each gene in the three × 11 cell type matrices.

**2.8.3 Joint Module Score**

The Seurat AddModuleScore function was used to calculate the joint module score of the five genes in all cells to quantitatively measure the overall activation level of each cell for that gene set. Cells were divided into two groups, ML5_High and ML5_Low, using the median module score as a threshold. The spatial distribution of cells with high and low scores was visualized using UMAP. The Wilcoxon rank-sum test was used to compare the score differences among the three groups, with a *p*-value <0.05 considered statistically significant.

**2.8.4 Pseudo-temporal trajectory analysis (Monocle3)**

A stratified subset of immune cells (T cells, B cells, myeloid cells, and mast cells, up to 2000 cells per group per type, totaling approximately 19,386 cells) was stratified from the full dataset. Seurat UMAP coordinates were reused to convert the data into Monocle3 CellDataSet objects. The `learn_graph` function (ncenter=100) was used to learn the cell development trajectory, and the pseudo-temporal sequence was sorted using the cell with the highest ML5gene_Score1 as the root node. The `plot_cells` function was used to plot cell type coloring maps, pseudo-temporal sequence coloring maps, group comparison maps, and expression gradient maps of the five target genes along the trajectory.

**2.8.5 Oxidative Stress Pathway Association Analysis**

A set of oxidative stress-related genes reported in the literature was collected and divided into five sub-modules: ROS generation, antioxidant, OS transcriptional regulation, hypoxia, and OS response. The AddModuleScore was used to calculate the module score for each module. The Pearson correlation coefficient was used to evaluate the correlation between ML5gene_Score1 and the scores of each oxidative stress module, and a correlation circle plot (ggcorrplot) was generated. Furthermore, the Pearson correlation coefficients between the five target genes and each gene in the oxidative stress gene set were calculated at the genome-wide level, and visualized as a heatmap.

An external public scRNA-seq dataset of nasal polyp tissues and control mucosa (Genome Sequence Archive，GSA，HRA000772) was used for cellular validation. The dataset included control, eCRSwNP, and neCRSwNP samples. Expression patterns of the identified genes were examined using UMAP feature plots, violin plots, dot plots, and heatmaps.

To assess coordinated activity, a five-gene module score was calculated using AddModuleScore. Pseudotime analysis was performed with Monocle3 on a stratified immune-cell subset to explore trajectory structure. In addition, correlations between the five-gene score and oxidative stress-related module scores were evaluated.

**2.9 Nasal tissue sample experiments**

**2.9.1 Quantitative Reverse Transcription Polymerase Chain Reaction (qRT-PCR)**

For qRT-PCR validation experiments, nasal mucosal tissue samples were collected from 20 patients with eCRSwNP, 17 patients with neCRSwNP, and 16 control subjects to validate the mRNA expression patterns of the identified key genes. CRSwNP samples were obtained from nasal polyp tissues during surgery, whereas control samples were obtained from relatively normal sinonasal mucosa of patients undergoing non-inflammatory procedures, including cerebrospinal fluid rhinorrhea repair, pituitary tumor surgery, lacrimal duct surgery, and septoplasty for deviated nasal septum. All tissue samples were immediately flash-frozen in liquid nitrogen after surgical resection and then transferred to a −80℃ freezer for later storage.

**2.9.1.1 Total RNA Extraction from Tissues**

Total RNA was extracted from tissues using TRIzol reagent (Invitrogen, USA), and the purity and concentration of RNA were detected using NanoDrop 2000 (Thermo Fisher Scientific). The specific operating procedures were consistent with RNA extraction and quality control in 2.2.3.1.

**2.9.1.2 Reverse Transcription**

Reverse transcription was performed using the Color Reverse Transcription Kit with gDNA Remover (catalog number A0010CGQ, EZBioscience). 500 ng of total RNA was used for reverse transcription from each sample. The specific operating steps are as follows: Calculate the sampling volume (x μL) according to the RNA concentration, add 2 μL of gDNA Remover, incubate at room temperature for 5 min to remove genomic DNA, and then immediately place on ice. After the reaction, add 5 μL of 4×RT Master Mix to the reaction system, and use ddH₂O to make up the total volume to 20 μL. Gently pipette to mix, and briefly centrifuge to collect the liquid at the bottom of the tube. The reverse transcription program is set as follows: 42℃ for 15 min; 95℃ for 30 s. After the reaction, obtain the cDNA product. Dilute the obtained cDNA 2-5 times with sterile ddH₂O for subsequent qPCR experiments.

**2.9.1.3 qPCR Amplification Reaction**

Quantitative real-time PCR amplification was performed using Color SYBR Green qPCR Master Mix (catalog number A0012-R2, EZBioscience). Primer treatment: Dilute the primer stock solution to 100 μM according to the instructions. Subsequently, 5 μL of forward primer and 5 μL of reverse primer were added to 40 μL of ddH₂O to prepare a primer working solution with a final concentration of 10 μM. The reaction mixture (10 μL per well) consisted of 5 μL Color SYBR Green qPCR Master Mix, 0.2 μL of primer mixture, 3.8 μL of ddH₂O, and 1 μL of diluted cDNA. After thorough mixing, 9 μL of the reaction mixture was added to a 384-well plate, followed by 1 μL of cDNA. The plate was sealed with a membrane and then gently centrifuged to remove air bubbles. Amplification was performed on a qPCR instrument using the following program: 95℃ pre-denaturation for 5 min; 40 cycles: 95℃ denaturation, 10 s annealing, 10 s 60℃ extension, and 30 s extension. After amplification, melting curve analysis was performed: the temperature was increased from 60℃ to 95℃ at a rate of 0.11℃/s to detect the specificity of the amplified products.

**2.9.1.4 Data Analysis**

Each sample was set up with triple replicates and normalized using the internal reference gene GUSB. One sample from the Control group was used as a calibration sample, and the sample without a template reaction served as a negative control. The calculation method is as follows: ΔCt = Ct_target − Ct_GUSB, where relative expression level is represented by 2^(-ΔCt) to indicate the relative expression level of the target gene mRNA in each sample.

**2.9.2 Immunohistochemical Staining (IHC)**

Paraffin-embedded nasal polyp tissue was prepared into 5 μm thick serial sections and mounted on anti-detachment slides. After dewaxing with xylene and hydration with graded ethanol, the sections underwent antigen retrieval in sodium citrate buffer (pH 6.0). Endogenous peroxidase activity was then blocked with 3% H₂O₂ and blocked with 5% goat serum.

The sections were incubated overnight at 4°C with the appropriate primary antibody. After washing with PBS, HRP-labeled secondary antibody was added, and the sections were incubated at 37°C for 30 min. DAB staining was performed, and hematoxylin was used to counterstain the cell nuclei. Finally, the sections were dehydrated with graded ethanol, cleared, and mounted. The sections were observed under an optical microscope (Olympus, Japan), and representative fields of view (200×, 400×) were acquired. Positively stained cells in ten different areas were counted under high power and quantified as cells per high-powered field (HPF, ×400). All slides images were reviewed by two independent investigators who were blinded to the study.

**2.9.3 Immunofluorescence (IF) Double Labeling Staining**

To clarify the cellular localization of key proteins in nasal polyp tissue, a double immunofluorescence staining method was used. The dewaxing, hydration, and antigen retrieval procedures for tissue sections were the same as for IHC. After blocking, sections were simultaneously incubated with the target protein antibody and the corresponding cell type marker antibody in a humidified chamber overnight at 4°C. After washing with PBS, the corresponding fluorescently labeled secondary antibody was added, and the sections were incubated at room temperature in the dark for 1 hour. Cell nuclei were stained with DAPI. After staining, slides were mounted with anti-fluorescence quenching mounting medium, and multi-channel fluorescence images were acquired under a laser confocal microscope (Leica TCS SP8, Germany). The co-localization of the target protein and cell markers was analyzed by image merging.

**2.10 Heatmap Visualization and Hierarchical Clustering**

Heatmaps were generated in R v4.5.1 using the pheatmap package (v1.0.13), whereas WGCNA-related dendrograms and module eigengene analyses were performed using the WGCNA package (v1.73). Hierarchical agglomerative clustering was applied where row or column clustering was required. The distance metrics and linkage methods used for individual analyses were selected according to the data type and visualization purpose, as described below.

**2.10.1 Global DEG expression heatmap (Figure 1B)**

Figure 1B displays two-way hierarchical clustering of the union of DEGs identified across the three pairwise comparisons among the control, eCRSwNP, and neCRSwNP groups. The heatmap was generated using the pheatmap package. Euclidean distance was used to calculate dissimilarities among both genes and samples, and complete linkage was used for hierarchical clustering. Gene-expression values were scaled by row before visualization.

**2.10.2 WGCNA-related analyses (Figure 3A–F)**

For gene module identification, a weighted adjacency matrix was first constructed from pairwise Pearson correlations between genes using the selected soft-thresholding power. The adjacency matrix was subsequently transformed into a topological overlap matrix (TOM). Genes were hierarchically clustered using topological overlap dissimilarity, defined as 1 − TOM, with average linkage. Modules were identified from the resulting gene dendrogram using the dynamic tree-cutting algorithm and were subsequently merged according to module eigengene similarity.

For module eigengene clustering, dissimilarity between module eigengenes was defined as 1 − Pearson correlation, and average-linkage hierarchical clustering was applied. Module–trait and module-immune-cell association heatmaps were displayed using the predefined module and trait order, without additional row or column clustering.

**2.10.3 Machine-learning feature-importance heatmap (Figure 5K)**

Hierarchical agglomerative clustering was applied to both rows and columns of the cross-algorithm feature-importance matrix. Pearson correlation-based distance, defined as 1 − r, was used as the dissimilarity metric, and complete linkage was used as the agglomeration method.

**2.10.4 Cell-type mean gene-expression heatmap (Figure 8S)**

The heatmap of mean expression levels of the five signature genes across groups and cell types was generated using two-way hierarchical agglomerative clustering. Pearson correlation-based distance, defined as 1 − r, was used for both rows and columns, with complete linkage as the agglomeration method.

**2.10.5 Oxidative stress and five-gene signature-score heatmaps (Figure 9K-P)**

For the correlation heatmap between the five-gene signature score and oxidative stress-related module scores, as well as the cell-type score heatmaps, hierarchical agglomerative clustering was applied to both rows and columns where applicable. Pearson correlation-based distance, defined as 1 − r, was used as the dissimilarity metric, and complete linkage was used as the agglomeration method.
