# Supplementary material for: Integration of transcriptomics and machine learning to explore inflammatory characteristics and key oxidative stress–related molecules in nasal polyps
Source: Front Immunol. 2026 Aug 26;17:1879295. doi: 10.3389/fimmu.2026.1879295 (PMC13553475; doi:10.3389/fimmu.2026.1879295)
Supplement: Supplementary file 2 [file SupplementaryFile2.docx]

**Supplementary Figures and Tables**

**Supplementary Figures**

**Supplementary Figure 1.** IPA canonical pathway, disease and biological function enrichment enrichment analysis among comparison groups. (A) The IPA classic signaling pathway enrichment analysis results of Control vs eCRSwNP; (B) The IPA disease and biological function enrichment analysis results of Control vs eCRSwNP; (C) The IPA classic signaling pathway enrichment analysis results of Control vs neCRSwNP; (D) The IPA analysis results of Control vs neCRSwNP; (E) The IPA classic signaling pathway enrichment analysis results of neCRSwNP vs eCRSwNP; (F) The IPA analysis results of neCRSwNP vs eCRSwNP. The color of the columns indicates the predicted direction of pathway activity (z-score): orange indicates pathway activation, blue indicates pathway inhibition, and gray indicates no clear activity prediction. The size of the bubbles represents the number of differentially expressed genes involved in the disease and biological function; the color of the bubbles indicates the predicted activity direction (z-score): orange indicates functional activation, blue indicates functional inhibition, and gray indicates no clear activity prediction.

**Supplementary Figure 2.** Violin plots showing immune cell infiltration profiles among comparison groups. (A–Q) The relative abundance distribution of 17 major immune cell subsets in the Control, neCRSwNP, and eCRSwNP groups; (R) The remaining low-abundance cells are combined into "Other" category for distribution. **P* < 0.05, ***P* < 0.01, ****P* < 0.001.

**Supplementary Figure 3.** Hyperparameter tuning and quality control of six machine learning models. **(A)** LASSO coefficient paths for selected genes across log(λ) values.
**(B)** Ridge regression coefficient paths (α = 0, pure L2 regularization). **(C)** Elastic net coefficient paths (α = 0.5). **(D)** Out-of-bag (OOB) error of random forest versus number of trees (ntree). **(E)** KNN classification boundaries in the first two principal component space. **(F)** Histogram of SVM decision values showing complete separation between control and eCRSwNP groups.

**Supplementary Tables**

**Supplementary Table 1**. **Demographic and clinical characteristics of the study participants.**

| **Variable** | **Control**  **(n=8)** | **neCRSwNP**  **(n=5)** | **eCRSwNP**  **(n=9)** | ***P value*** |
| --- | --- | --- | --- | --- |
| Age, years | 39.9±12.9 | 42.4±12.3 | 48.9±17.9 | 0.528 |
| Male, n (%) | 7 (87.5) | 4 (80.0) | 6 (66.7) | 0.809 |
| Asthma, n (%) | 0 (0) | 0 (0) | 3 (33.3) | 0.149 |
| Blood eosinophils, % | 2.31±1.15 | 3.18±1.73 | 9.44±6.07 | 0.004 |
| Blood eosinophils, ×10⁹/L | 0.13±0.06 | 0.21±0.13 | 0.55±0.37 | 0.006 |
| Tissue eosinophils, % | 0.94±0.84 | 6.40±1.34 | 30.44±7.23 | <0.001 |
| Tissue eosinophils, cells/HPF | 0.9±0.8 | 4.6±1.1 | 60.6±17.5 | <0.001 |

neCRSwNP, non-eosinophilic chronic rhinosinusitis with nasal polyps; eCRSwNP, eosinophilic chronic rhinosinusitis with nasal polyps; HPF, high-power field.

Supplementary Table 2.The primer sequences used in qRT-PCR

| **Gene name** |  | **Primer** |
| --- | --- | --- |
| GUSB | Forward | GTCTGCGGCATTTTGTCGG |
|  | Reverse | CACACGATGGCATAGGAATGG |
| NCF2 | Forward | GAGGCTCACCGTGTGCTATT |
|  | Reverse | GGCCCAGTTATCATTGCCCT |
| RAC2 | Forward | CTCCTATCCACAGACGGACG |
|  | Reverse | TCTCGATGGTGTCCTTGTCG |
| NCF4 | Forward | CGGGCCGAGAGTGACTT |
|  | Reverse | AAAGCATGGAACTGGCGGTA |
| HIF1A | Forward | GGCGCGAACGACAAGAAA |
|  | Reverse | TGGGTGAGGAATGGGTTCAC |
| SELP | Forward | GGACTGTGTTCATCCGCTCA |
|  | Reverse | AGAGCAATCCATGCTTCCGT |

Supplementary Table 3. Experimental antibody information

| **Antibody** | **Item number** | **Company** |
| --- | --- | --- |
| CD3 | MAB-0740 | Maixin |
| CD8 | MAB-1031 | Maixin |
| CD138 | MAB-0200 | Maixin |
| CD20 | Kit-0001 | Maixin |
| CD163 | MAB-0869 | Maixin |
| CD68 | Kit-0026 | Maixin |
| MPO | MAB-0853 | Maixin |
| CD34 | Kit-0004 | Maixin |
| NCF2 | 15551-1-AP | Proteintech |
| NCF4 | 14648-1-AP | Proteintech |
| SELP | 83947-5-RR | Proteintech |
| RAC2 | 10735-1-AP | Proteintech |
| HIF1A | 20960-1-AP | Proteintech |
| TPSAB1 | 66174-1-Ig | Proteintech |
| CD11c | 60258-1-Ig | Proteintech |
| Ribonuclease3 | EM1801-17 | HUABIO |

Supplementary Table 4. The IPA canonical pathway enrichment results of DEGs between the control and eCRSwNP groups

| **Ingenuity Canonical Pathways** | **-log10(*p*-value)** | **Ratio** | **z-score** |
| --- | --- | --- | --- |
| Phagosome Formation | 38.8 | 0.158 | 9.798 |
| Pathogen Induced Cytokine Storm Signaling Pathway | 38.1 | 0.224 | 7.5 |
| Immunoregulatory interactions between a Lymphoid and a non-Lymphoid cell | 37.2 | 0.285 | 7.81 |
| Neutrophil degranulation | 29.2 | 0.164 | 8.832 |
| Th2 Pathway | 28.8 | 0.323 | 4.542 |
| Molecular Mechanisms of Cancer | 26.4 | 0.12 | 9.39 |
| S100 Family Signaling Pathway | 22.2 | 0.117 | 8.944 |
| Natural Killer Cell Signaling | 21.3 | 0.219 | 5.096 |
| FAK Signaling | 21.3 | 0.11 | 9.113 |
| Th1 Pathway | 20 | 0.28 | 5.477 |
| CREB Signaling in Neurons | 20 | 0.123 | 8.128 |
| Class A/1 (Rhodopsin-like receptors) | 19.4 | 0.16 | 7.28 |
| Leukocyte Extravasation Signaling | 18.3 | 0.205 | 4.802 |
| Breast Cancer Regulation by Stathmin1 | 17.5 | 0.117 | 7.632 |
| Lung Ionic Balance Signaling Pathway | 17.2 | 0.124 | 7.685 |
| G-Protein Coupled Receptor Signaling | 16.9 | 0.108 | 7.778 |
| Neutrophil Extracellular Trap Signaling Pathway | 16.4 | 0.137 | 3.539 |
| Role of Pattern Recognition Receptors in Recognition of Bacteria and Viruses | 15.3 | 0.215 | 3.357 |
| Role of Osteoclasts in Rheumatoid Arthritis Signaling Pathway | 15.1 | 0.149 | 5.578 |

Supplementary Table 5. The IPA canonical pathway enrichment results of DEGs between the control and neCRSwNP groups

| **Ingenuity Canonical Pathways** | **-log10(*p*-value)** | **Ratio** | **z-score** |
| --- | --- | --- | --- |
| Pathogen Induced Cytokine Storm Signaling Pathway | 13.4 | 0.0941 | 5 |
| Phagosome Formation | 10.6 | 0.0605 | 6 |
| Tumor Microenvironment Pathway | 9.11 | 0.107 | 4.243 |
| Molecular Mechanisms of Cancer | 8.32 | 0.0498 | 6.245 |
| Class A/1 (Rhodopsin-like receptors) | 7.24 | 0.0693 | 4.796 |
| S100 Family Signaling Pathway | 7.22 | 0.0491 | 5.745 |
| Irritable Bowel Syndrome Signaling Pathway | 7.19 | 0.0745 | 3.273 |
| Immunoregulatory interactions between a Lymphoid and a non-Lymphoid cell | 6.33 | 0.0794 | 4.123 |
| Lung Ionic Balance Signaling Pathway | 6.06 | 0.0531 | 4.811 |
| Cardiac Hypertrophy Signaling (Enhanced) | 5.7 | 0.0508 | 4.146 |
| Role of Osteoclasts in Rheumatoid Arthritis Signaling Pathway | 5.61 | 0.0644 | 3.357 |
| FAK Signaling | 5.41 | 0.0425 | 5.303 |
| G-Protein Coupled Receptor Signaling | 5.26 | 0.0445 | 4.017 |
| Cellular Effects of Sildenafil (Viagra) | 5.09 | 0.0444 | -3.9 |
| CREB Signaling in Neurons | 4.71 | 0.0449 | 4.707 |
| BBSome Signaling Pathway | 4.49 | 0.0476 | 4.69 |
| IL-17 Signaling | 4.44 | 0.0707 | 3.317 |
| Breast Cancer Regulation by Stathmin1 | 4.42 | 0.0441 | 4.491 |
| G alpha (i) signalling events | 4.03 | 0.0579 | 3.873 |
| RAF/MAP kinase cascade | 3.98 | 0.0573 | 3.742 |
| Pulmonary Fibrosis Idiopathic Signaling Pathway | 3.5 | 0.0497 | 4 |
| Neutrophil degranulation | 2.14 | 0.0356 | 4.123 |
| Autism Signaling Pathway | 2.01 | 0.0399 | 3.317 |

Supplementary Table 6. The IPA canonical pathway enrichment results of DEGs between the neCRSwNP vs eCRSwNP groups

| **Ingenuity Canonical Pathways** | **-log10(*p*-value)** | **Ratio** | **z-score** |
| --- | --- | --- | --- |
| Phagosome Formation | 26.4 | 0.112 | 8.367 |
| CREB Signaling in Neurons | 23.1 | 0.111 | 7.81 |
| Molecular Mechanisms of Cancer | 22.4 | 0.0937 | 8.66 |
| Lung Ionic Balance Signaling Pathway | 19.8 | 0.112 | 7.285 |
| Breast Cancer Regulation by Stathmin1 | 19.4 | 0.103 | 7.353 |
| Neutrophil degranulation | 18.9 | 0.113 | 7.348 |
| G-Protein Coupled Receptor Signaling | 18.9 | 0.0948 | 7.181 |
| S100 Family Signaling Pathway | 17.7 | 0.0889 | 7.62 |
| Class A/1 (Rhodopsin-like receptors) | 15.1 | 0.12 | 6.325 |
| FAK Signaling | 14.7 | 0.0789 | 7.81 |
| BBSome Signaling Pathway | 14.6 | 0.0994 | 6.708 |
| Immunoregulatory interactions between a Lymphoid and a non-Lymphoid cell | 12.4 | 0.136 | 5.385 |
| G alpha (q) signalling events | 8.16 | 0.12 | 4.583 |

**Supplementary Table 7. Raw feature importance values or regression coefficients derived from six machine learning models and SHAP.**

| **Gene** | **LASSO_**  **Coef** | **Ridge_**  **Coef** | **ElasticNet_**  **Coef** | **RF_**  **Imp** | **KNN_**  **Imp** | **SVM_**  **Imp** | **SHAP_**  **Imp** |
| --- | --- | --- | --- | --- | --- | --- | --- |
| HIF1A | 3.745069 | 0.065475 | 2.757521 | 6.196031 | 0.058824 | 0.005443 | 1.036912 |
| RAC2 | 3.707939 | 0.059221 | 1.419585 | 4.985317 | 0.042017 | 0.005506 | 0.893147 |
| NCF2 | 2.15198 | 0.050242 | 1.366131 | 5.536808 | 0.02521 | 0.005753 | 0.553423 |
| SELP | 1.382534 | 0.054984 | 1.179852 | 1.829136 | 0.067227 | 0.008463 | 0.450388 |
| NCF4 | 0 | 0.055961 | 1.310133 | 6.623167 | 0.02521 | 0.004482 | 0.229094 |
| TYROBP | 0 | 0.041286 | 0.87034 | 5.128356 | 0.02521 | 0.003003 | 0.12648 |
| HMOX1 | 0 | 0.042382 | 0.389287 | 6.104949 | 0.008403 | 0.004707 | 0.066814 |
| SYK | 0 | 0.050221 | 0.208762 | 3.282497 | 0.008403 | 0.003232 | 0.048057 |
| PTPRC | 0 | 0.047801 | 0.131283 | 4.027792 | 0 | 0.003983 | 0.031783 |
| SELL | 0 | 0.044328 | 0.117212 | 4.031446 | 0.016807 | 0.003069 | 0.028147 |
| CCL2 | 0 | 0.047917 | 0.076649 | 3.893704 | 0 | 0.003789 | 0.022322 |
| ITGB2 | 0 | 0.047547 | 0.055498 | 1.566536 | 0.016807 | 0.002823 | 0.021169 |
| CYBB | 0 | 0.047886 | 0.009157 | 3.124213 | 0.008403 | 0.005359 | 0.013441 |
| ICAM1 | 0 | 0.051342 | 0 | 0 | 0 | 0.007237 | 0.01316 |
| VCAM1 | 0 | 0.046695 | 0 | 1.446199 | 0 | 0.005464 | 0.011502 |
| CSF1R | 0 | 0.045757 | 0 | 0.368134 | 0.008403 | 0.005375 | 0.011447 |
| PECAM1 | 0 | 0.043663 | 0 | -0.92639 | 0.016807 | 0.004089 | 0.010484 |
| CXCR4 | 0 | 0.026292 | 0 | -0.5005 | 0 | 0.003215 | 0.004389 |

Coef, coefficient; Imp, importance.

**Supplementary Table 8.** **Standardized feature importance values or regression coefficients derived from six machine learning models and SHAP.**

| **Gene** | **LASSO_IpN** | **Ridge_**  **IpN** | **ElasticNet_IpN** | **RF_**  **IpN** | **KNN_**  **IpN** | **SVM_**  **IpN** | **SHAP_**  **IpN** | **Total_**  **IpN** |
| --- | --- | --- | --- | --- | --- | --- | --- | --- |
| HIF1A | 1 | 1 | 1 | 0.935 | 0.875 | 0.643 | 1 | 6.453 |
| RAC2 | 0.990 | 0.904 | 0.514 | 0.752 | 0.625 | 0.650 | 0.861 | 5.298 |
| SELP | 0.369 | 0.839 | 0.427 | 0.276 | 1 | 1 | 0.434 | 4.347 |
| NCF2 | 0.574 | 0.767 | 0.495 | 0.835 | 0.375 | 0.679 | 0.533 | 4.261 |
| NCF4 | 0 | 0.854 | 0.475 | 1 | 0.375 | 0.529 | 0.220 | 3.455 |
| TYROBP | 0 | 0.630 | 0.315 | 0.774 | 0.375 | 0.354 | 0.121 | 2.572 |
| HMOX1 | 0 | 0.643 | 0.141 | 0.921 | 0.125 | 0.556 | 0.064 | 2.455 |
| CYBB | 0 | 0.731 | 0.003 | 0.471 | 0.125 | 0.633 | 0.012 | 1.977 |
| SELL | 0 | 0.677 | 0.043 | 0.608 | 0.25 | 0.362 | 0.027 | 1.967 |
| SYK | 0 | 0.767 | 0.076 | 0.495 | 0.125 | 0.381 | 0.046 | 1.891 |
| PTPRC | 0 | 0.730 | 0.048 | 0.608 | 0 | 0.470 | 0.030 | 1.887 |
| CCL2 | 0 | 0.731 | 0.028 | 0.587 | 0 | 0.447 | 0.021 | 1.816 |
| ICAM1 | 0 | 0.784 | 0 | 0 | 0 | 0.855 | 0.012 | 1.651 |
| VCAM1 | 0 | 0.713 | 0 | 0.218 | 0 | 0.645 | 0.011 | 1.588 |
| ITGB2 | 0 | 0.726 | 0.020 | 0.236 | 0.25 | 0.333 | 0.020 | 1.586 |
| CSF1R | 0 | 0.698 | 0 | 0.055 | 0.125 | 0.635 | 0.011 | 1.525 |
| PECAM1 | 0 | 0.666 | 0 | -0.138 | 0.25 | 0.483 | 0.010 | 1.270 |
| CXCR4 | 0 | 0.401 | 0 | -0.075 | 0 | 0.379 | 0.004 | 0.710 |

IpN, normalized importance value.

**Supplementary Table 9. Results of single-gene ROC analysis for the five core signature genes.**

| **Gene** | **AUC** | **95% CI** | **Sensitivity** | **Specificity** |
| --- | --- | --- | --- | --- |
| NCF2 | 0.991 | 0.968–1.000 | 100% | 87.5% |
| RAC2 | 0.949 | 0.856–1.000 | 100% | 75.0% |
| NCF4 | 0.940 | 0.846–1.000 | 100% | 62.5% |
| SELP | 0.889 | 0.671–1.000 | 88.9% | 87.5% |
| HIF1A | 0.863 | 0.710–1.000 | 88.9% | 62.5% |

AUC, area under the curve; CI, confidence interval.
